# Supplementary material for: Wide-range soft anisotropic thermistor with a direct wireless radio frequency interface
Source: Nat Commun. 2024 Jan 11;15:452. doi: 10.1038/s41467-024-44735-z (PMC10781794; doi:10.1038/s41467-024-44735-z)
Supplement: Supplementary file 1 — Supplementary Information [file 41467_2024_44735_MOESM1_ESM.pdf]

Supplementary Material for “Wide-Range Soft Anisotropic Thermistor with a Direct Wireless Radio Frequency Interface”

Mahmoud Wagih*, Junjie Shi, Menglong Li, Abiodun Komolafe, Thomas Whittaker, Johannes Schneider, Shanmugam Kumar, William Whittow, Steve Beeby

Correspondence: mahmoud.wagih@glasgow.ac.uk

This supplementary file includes information and detailed figures about:

1. Comparing the DC and RF response of the proposed thermistor
2. Radiation From a Microstrip Patch on a Low-Resistivity Thermistor Substrate.
3. Fabrication, Processing, And SEM Micrographs.
4. DC Properties and Repeatability.
5. 40% CF Composite Repeatability and Full Response.
6. Thermistor’s Response Under Deformation
7. Cross-Sensitivity in Varying Humidities
8. Thermistor’s TGA and DSC Response
9. Composite Coefficient of Thermal Expansion
10. Temperature Cycling and Thermistor Anisotropy.
11. Broadband RF Permittivity and Loss Extraction from a Transmission Line.
12. Band-Specific RF Conductivity Measurements.
13. RF Anisotropy’s Impact on the In-Plane Material Properties
14. Flexible And Printable Temperature Sensing Materials Comparison.
15. Microwave Resonator Dimensions and Broadband S-Parameters Response.
16. Resonator 2 Design and Dimensions.
17. Comparison With Previous RF Temperature Sensors.
18. Patch Antenna Dimensions and Near-Field Characterisation.
19. Anechoic Radiation Pattern Measurements.
20. Far-Field Temperature Sensitivity Characterisation
21. RFID RSSI Repeatability Across Channels

Supplementary Note 1. Comparing the DC and RF response of the proposed thermistor

As illustrated qualitatively in Figure 1**,** the thermistor's resistance change in response to temperature is spread over a wider temperature range at higher frequencies. Thus, reading the resistivity at RF, through the losses in a resonant or non-resonant RF component's transmission response, would allow the proposed thermistor to be read over a significantly wider range than its DC counterparts.

Supplementary Figure 1 shows the measured sheet resistance of the 40 wt.% CF composite at DC, measured using a multi-meter (see the Methods section), and the RF response at 2.4 GHz, measured using a microstrip line (as detailed in Supplementary Note 11, and the Methods section). As detailed later in the Supplementary documents and in the Methods section, the RF measurements were limited to 230°C, due to the temperature handling of the coaxial cables.

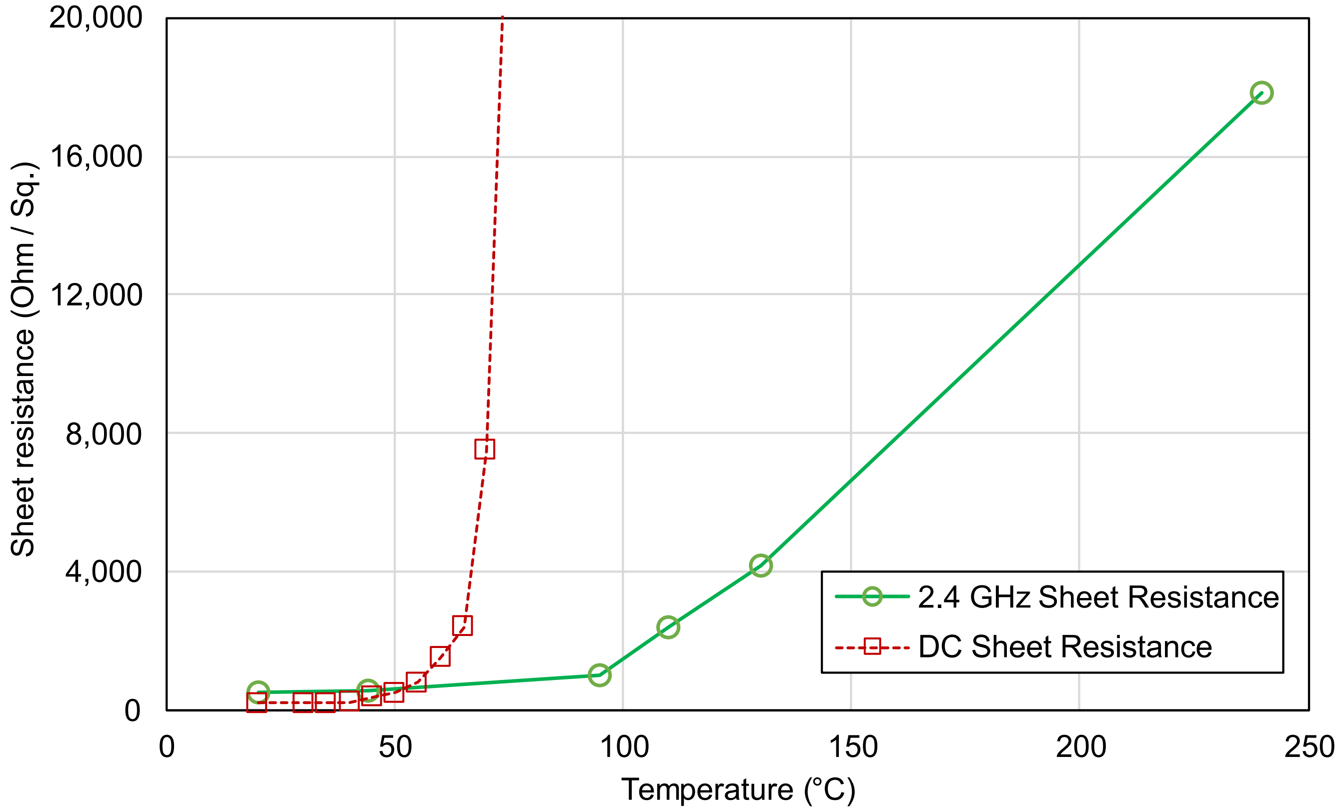

Supplementary Figure 1. The quantitative difference in the resistance's response between DC and RF readouts, showing a temperature sensing range in excess of 200°C with the RF readout.

Supplementary Note 2: Radiation from a Microstrip Patch on a Low-Resistivity Thermistor Substrate.

The response shown in Figure 1(d) and (e) was simulated using CST Microwave Studio, based on the dimensions of the patch antenna characterised in Figure 4, and detailed in Supplementary Note 11. The antenna was simulated based on two representative substrate dielectric properties:

- For room temperature: $\sigma_x = \sigma_y = 10\ S/m\ ;\quad \sigma_z = 0.05\ S/m$
- For high temperature: $\sigma_x = \sigma_y = 1 \times 10^{-8}\ S/m\ ;\quad \sigma_z = 0.01\ S/m$

The simulated E-fields and model are shown in supplementary Figure 2.

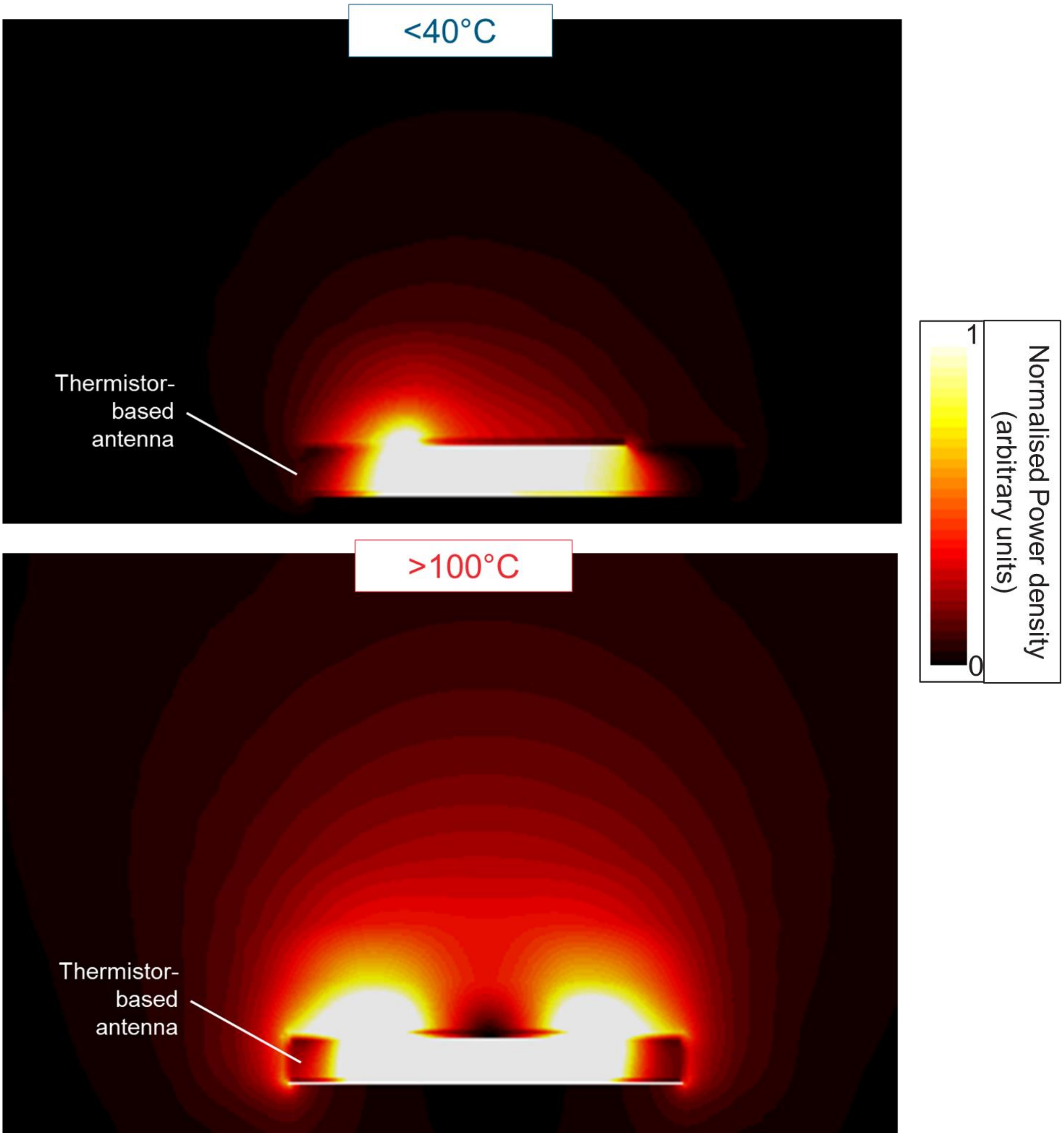

Supplementary Figure 2. *E*-field power density around a microstrip patch antenna based on the proposed thermistor composite as a substrate.

Supplementary Note 3: Fabrication, Processing, and SEM Micrographs

The fabrication process is shown in Supplementary Figure 3 As described in the Methods section, the mixed PDMS/CF/IPA solution is heated to evaporate the IPA before being vacuumed in the 3D moulds, used to shape the sensor. Following a 120-minutes curing process at 80°C, the composite was left to dry and stabilize at room temperature for 24 hours, until no further electrical resistance changes were observed. Silver electrodes were added to the samples for the DC measurements, to ensure repeatability and avoid any variations in the contact resistance.

For the RF measurements, as the dielectric properties are the most interesting and responsible for modulating the amplitude of the signals, the contact resistance tolerances are negligible as the capacitive coupling of the electric fields through the Polymer Matrix Composite (PMC) can be maintained.

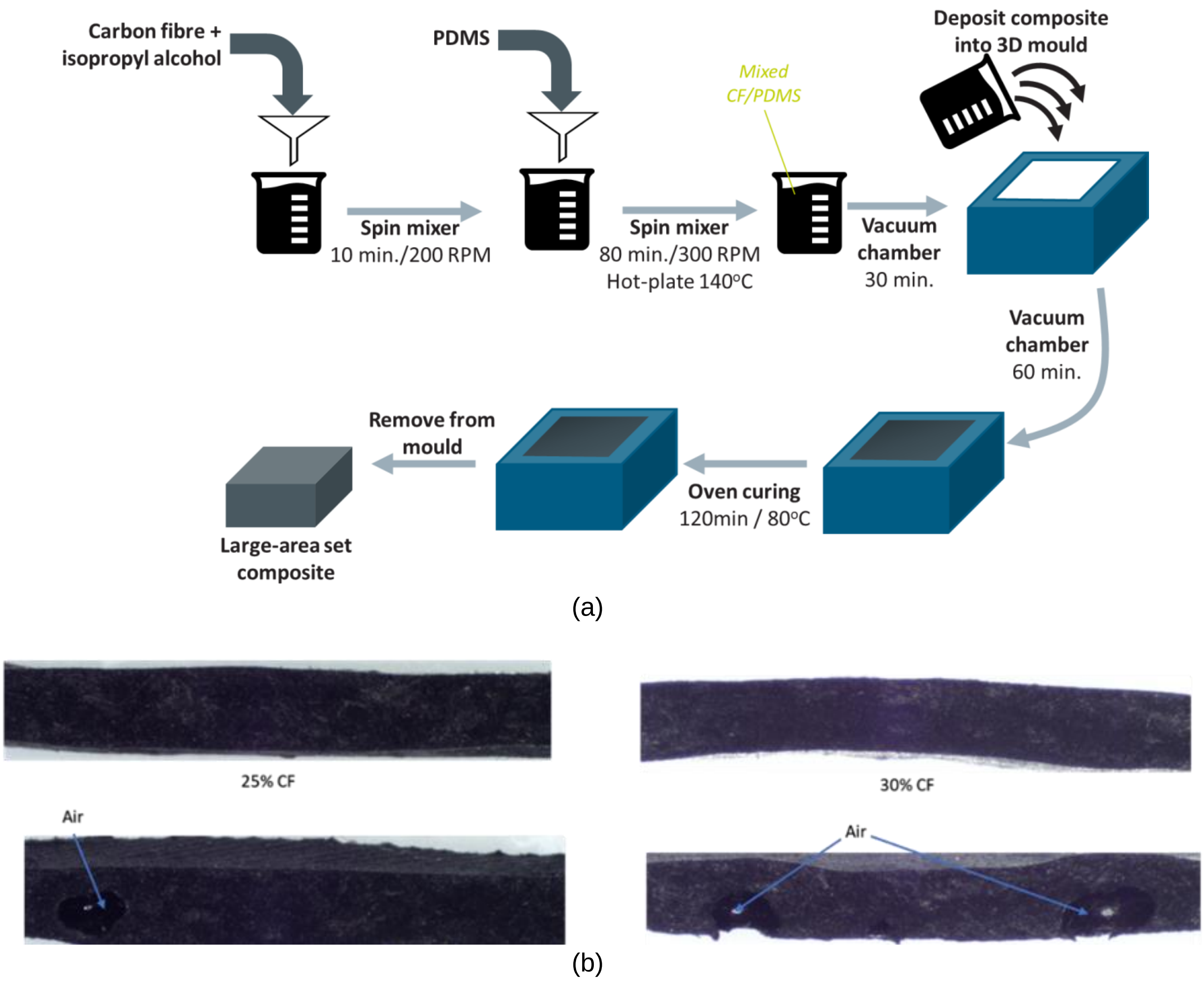

Supplementary Figure 3. (a) Fabrication process of the composite thermistor. (b) Photographs of the PMC thermistor with and without the vacuum process; the observed air pockets are eliminated through the vacuum process described in the Methods.

The PMC can be seen in the SEM micrographs in Supplementary Figure 4 for different magnifications. The sample was cryogenically fractured, where the conductive CFs closely overlap each other at room temperature, enabling them to maintain a relatively high electrical conductivity. In the 500× magnification micrograph, it can be observed that the highest concentration of the CFs is in-plane, with sporadic distribution of the fibres out-of-plane. This is caused by the deposition mechanism of the CF/PDMS composite in Supplementary Figure 3, where the fibres align in the direction of the plane they are being poured into. As a result, the conductive fibres take a layered structure, which is evident in the micrographs in Supplementary Figure 4. Consequently, the thermistor exhibits an anisotropic resistivity and TCR, with a significantly higher in-plane conductivity compared to out-of-plane, as measured in Supplementary Note 4.

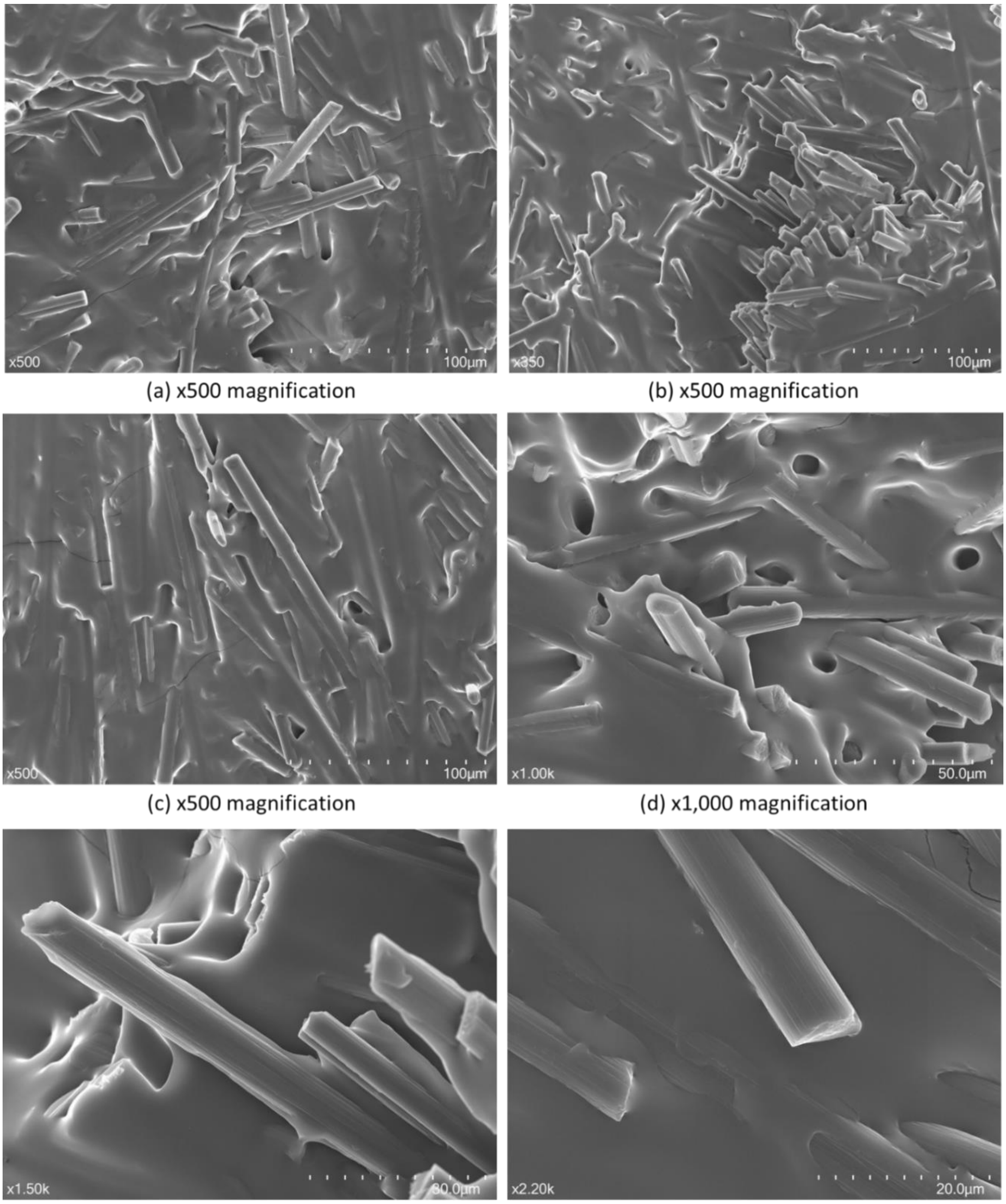

(a) x500 magnification (b) x500 magnification

(c) x500 magnification (d) x1,000 magnification

(e) x1,500 magnification (f) x2,200 magnification

Supplementary Figure 4. Scanning electron microscope (SEM) images of the 35% CF/65% PDMS w.t. PMC thermistor for varying magnifications: (a), (b), (c) 500x magnification taken across different cross-sections of the composite; (d) 1,000x magnification showing the fibres' alignment; (e) 1,500x magnification showing an individual fractured fibre; (f) 2,200x magnification showing the fibre's cross-section.

The SEM micrographs of the four PMCs prepared, for 25 wt.%, 30 wt.%, 35 wt.%, and 40 wt.% CF loadings are shown in Supplementary Figure 5. From the micrographs, the higher density of the CFs can be visualized, which translates to the improved electrical conductivity. Furthermore, the close horizontal alignment is also observed along with the layered structure of the composite. Due to the thickness of the sample, the micrographs shown in Supplementary Figure 5 were taken from a sample cut using a sharp blade, and were not cryogenically fractured.

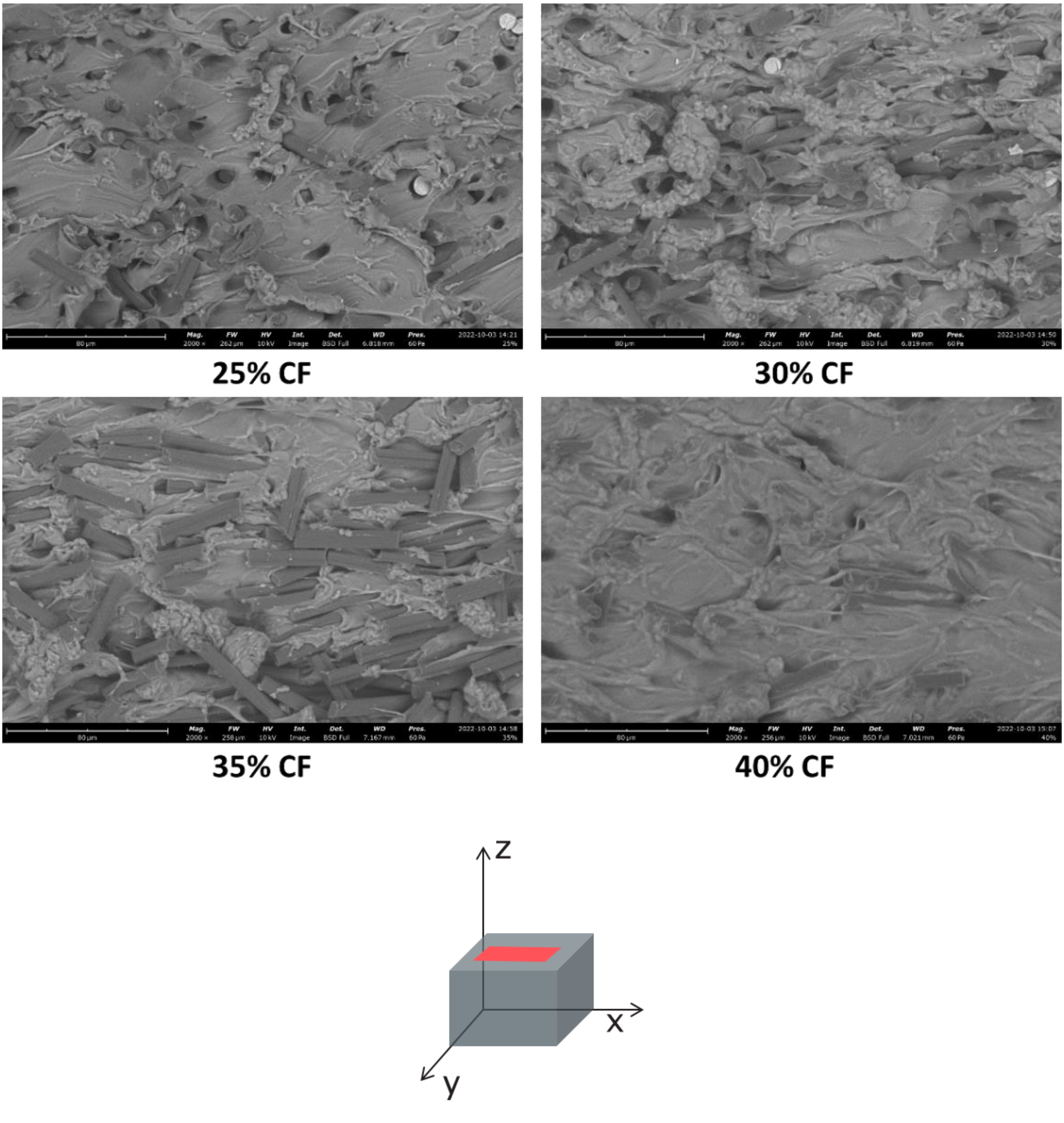

Supplementary Figure 5. SEM images of the PMC thermistor for varying CF/PDMS ratios, in the XY plane, at 2000× magnification; the red marking indicates the plane over which the SEMs were taken.

The layered structure of the composite is shown in Supplementary Figure 6. The SEMs show two cross sections, along the x-axis, where the CFs are aligned, and along the y-axis, perpendicular to the CFs' alignment. These are shown in Supplementary Figure 5(a) and (b) respectively. Together with the measured electrical response, at DC and RF, in Figure 2(g) and (h), the alignment of the CFs in the casting direction is evident and the cause of the observed anisotropy.

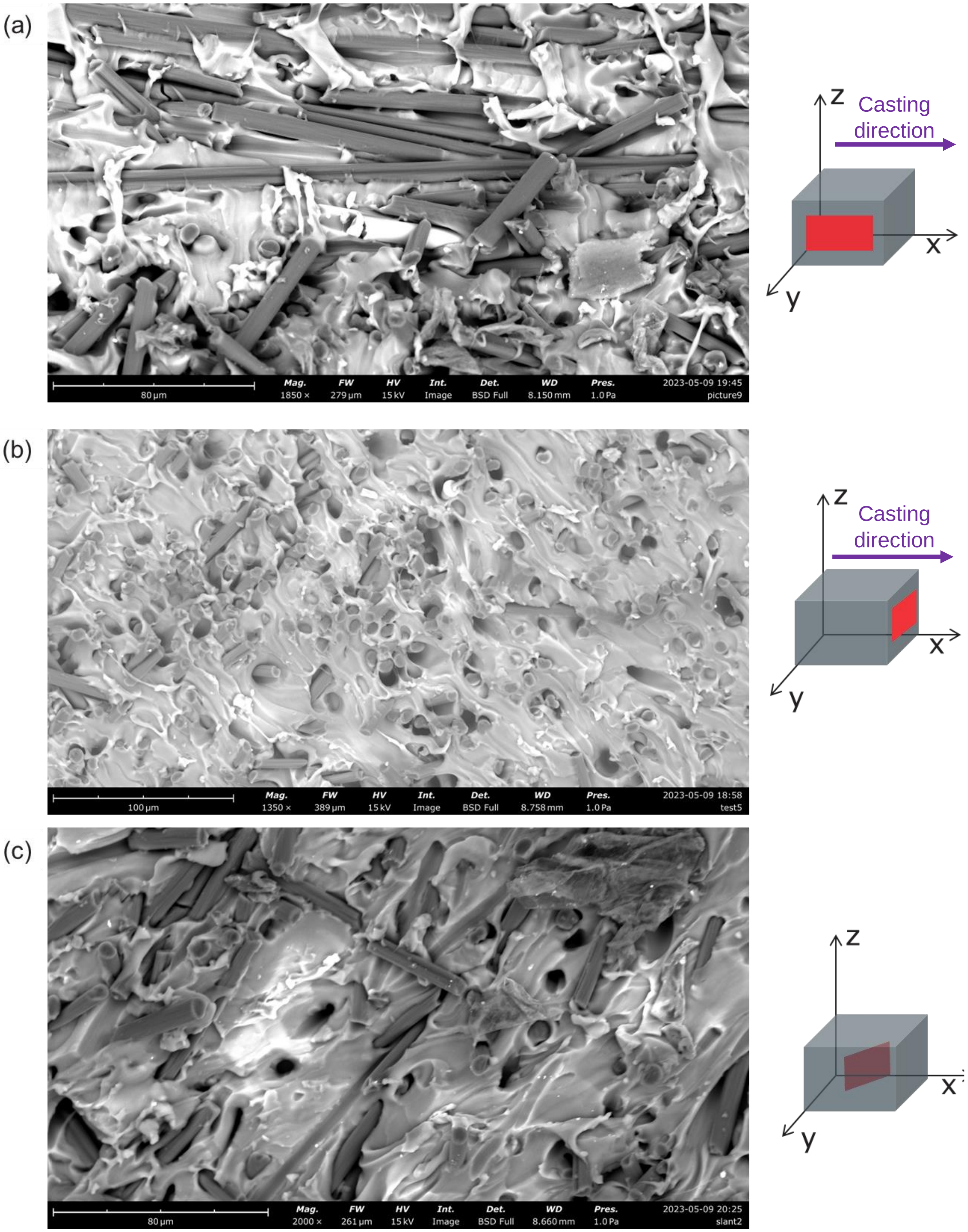

Supplementary Figure 6. SEM images of the composite for: (a) the x direction; (b) y direction; (c) diagonal cut in the XYZ plane; the red marking indicates the plane over which the SEMs were taken.

Supplementary Note 4: DC Properties and Repeatability

Supplementary Figure 7 shows the measured DC sheet resistance of the material for the four CF:PDMS ratios. The observed variation is due to the high roughness of the surface combined with the softness of the composite. The mean sheet resistance values were used in Figure 2(g). The measurement setup is shown in Supplementary Figure 8, with the spring-loaded pins landed on the sample.

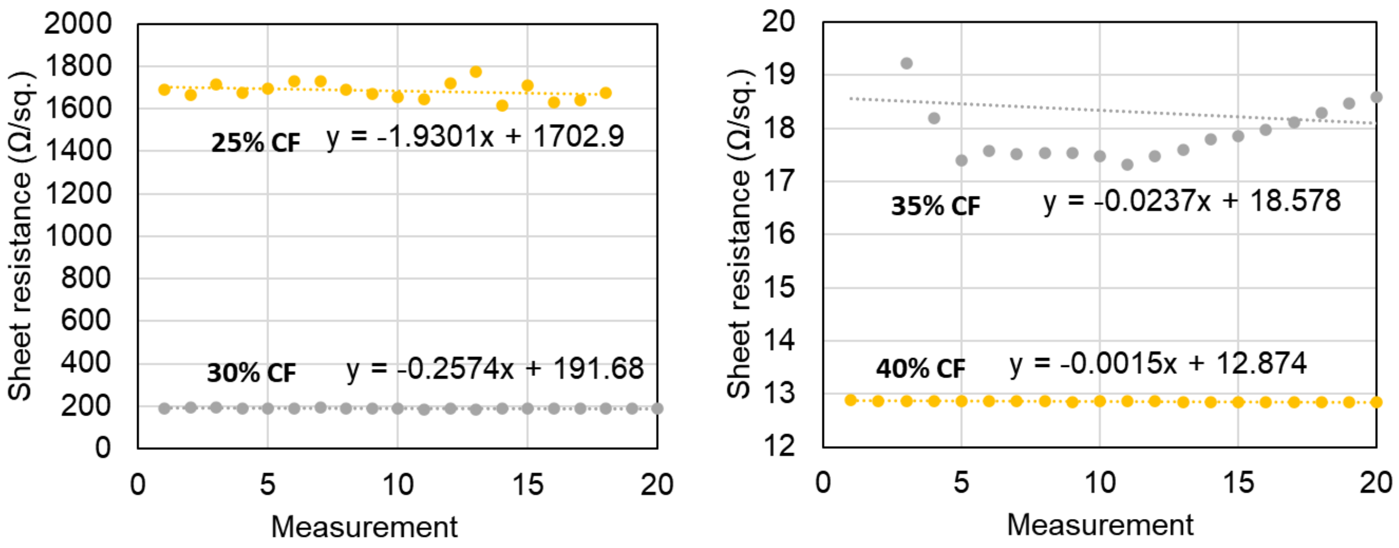

Supplementary Figure 7. Measured sheet resistance of the composite using the four-probe setup over 20 takes, for all CF/PDMS ratios formulated.

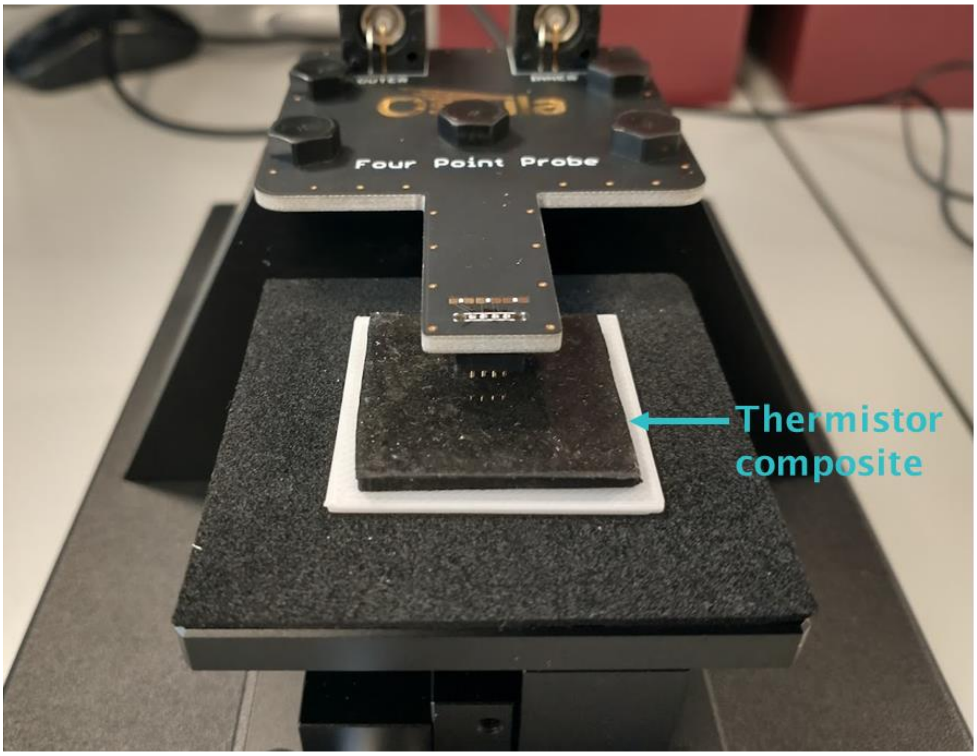

Supplementary Figure 8. Measurement setup of the composite using the four-point probe.

Supplementary Note 5: Repeatability and the 40% CF Full Response

The repeatability of the same thermistor's response over different measurements is shown in Supplementary Figure 9, for the 40% CF composite.

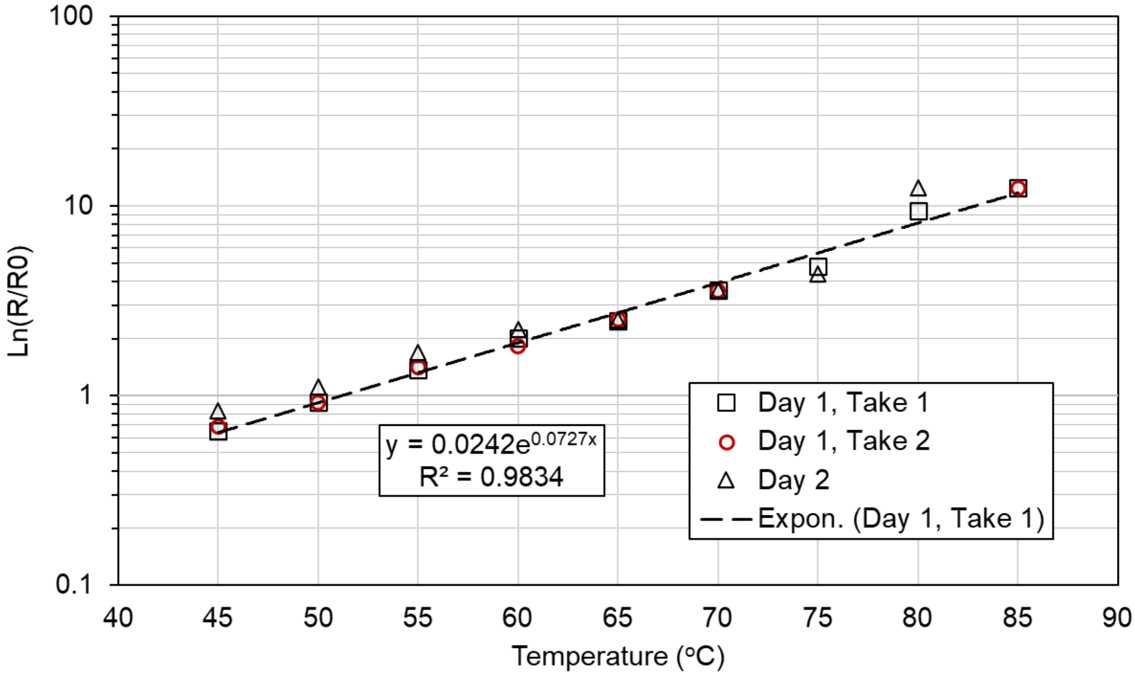

Supplementary Figure 9. Repeatability of the 40% CF composite's thermistor response, shown on a logarithmic plot using the natural logarithm of the normalised resistance change.

The bulk "apparent" resistivity values of the four PMC formulations are shown in Supplementary Table 1; these values are calculated assuming the material is isotropic and are therefore distinct form the anisotropic resistivity of the composite, both in-plane and out-of-plane. For the DC response, an increase in the CF loading directly increases the conductivity. On the other hand, the increase in the DC conductivity relates inversely to the stretchability of the material. As observed in Supplementary Table 1, the 40 wt.% CF/PDMS composite has the lowest stretchability, of 40 wt.%, which is due to the stiffness of the CF compared to the PDMS binder.

The maximum TCR is observed in the 20 to 50°C range for the 30 wt.% CF/PDMS composite, which is due to the trade-off between the room temperature electrical conductivity, increased by increasing the CF loading, and the ability of the expanding polymer matrix (the PDMS) to separate the CFs and increase the observed resistance change. However, based on Supplementary Figure 8, the 40 wt.% CF/PDMS composite achieves a TCR over 2000°C^{-1} when evaluated up to 85°C.

Supplementary Table 1. Summary of the DC and mechanical parameters of the PMCs for different CF/PDMS ratios

CF% by w.t.	Apparent bulk resistivity at 24°C (Ω/m)	Maximum strain	DC TCR, in-plane, between 20 and 50°C (°C^{-1})
**25%**	583	96%	1804.4
**30%**	362	70%	2685
**35%**	258	51%	25.36
**40%**	132	40%	0.2327

Supplementary Note 6: Thermistor's Response Under Deformation

The composite is resilient to cyclic bending and stretching, and has been tested over more cycles (1,000) compared to state-of-the-art soft thermistors, which have only been tested for 200 stretching cycles [36]. The effect of simultaneous heating and deformation (both stretching and bending) is presented.

Supplementary Figure 12(a) shows the temperature-resistance relation, for the unstretched pristine thermistor, and under 20.8% strain. As depicted in the diagram, the thermistor is stretched using a clamped fixture and the length between the two measurement electrodes is measured and used to calculate the strain. The observed shift the in the thermistor's response to higher temperatures is attributed to the compression in the z-axis. The strain increases the room-temperature resistance, in-plane, while decreasing the out-of-plane resistance, as seen in Supplementary Figure 12(b). This causes the sample to exhibit a higher conductivity at higher temperatures, which would otherwise separate all the in-plane contacts between the conductive CFs.

In most wearable applications, however, the components are mostly subjected to bending (which induces compressive and tensile strain [S1]). Supplementary Figure (c) shows that for two bending radii, 1.75 cm and 3.25 cm, the observed change in the thermistor's response is minimal, unlike stretching. Therefore, unless a compensation mechanism is included for independently measuring strain, the sensing functionality of the material is restricted to bendable, but not highly stretchable, applications. This limitation also arises from the stretchability of the RF circuit traces, which might not be implemented using stretchable conductors.

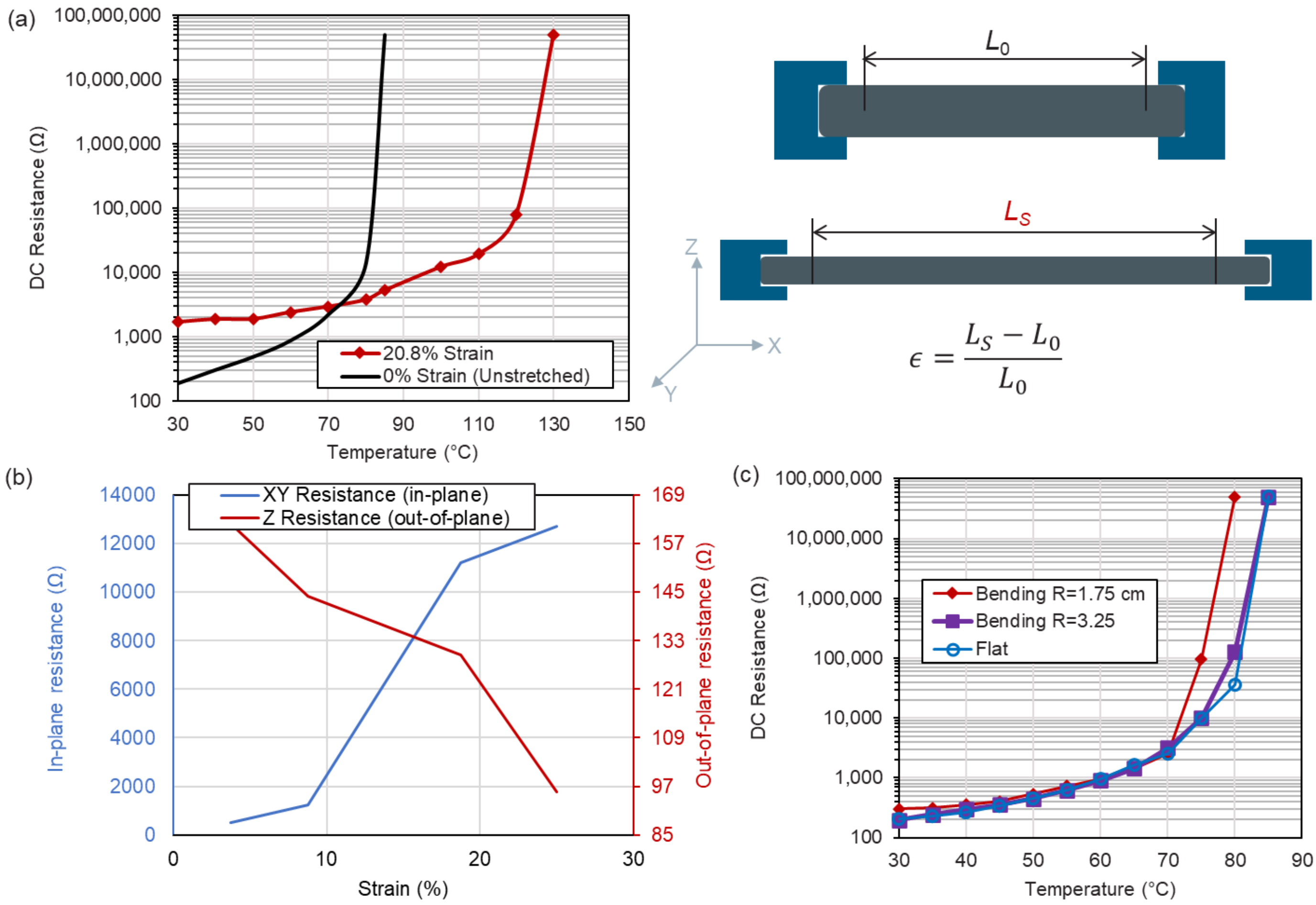

Supplementary Figure 12. Effect of strain and bending on the composite's resistance: (a) Measured resistance of the composite over temperature when linearly stretched; (b) room temperature resistance, in-plane and out-of-plane, under for various strain values; (c) the measured temperature-resistance relation for different bending radii.

Supplementary Note 7: Cross-Sensitivity in Varying Humidities

As PDMS is sensitive to humidity, the cross sensitivity to humidity has been investigated at a fixed temperature. Supplementary Figure 10 shows how the resistance change in response to humidity is under 25%. As previously seen in Figure 2(c), the material's temperature sensitivity results in a TCR between 40 and 1000, which is several orders of magnitude higher than the sensitivity to humidity.

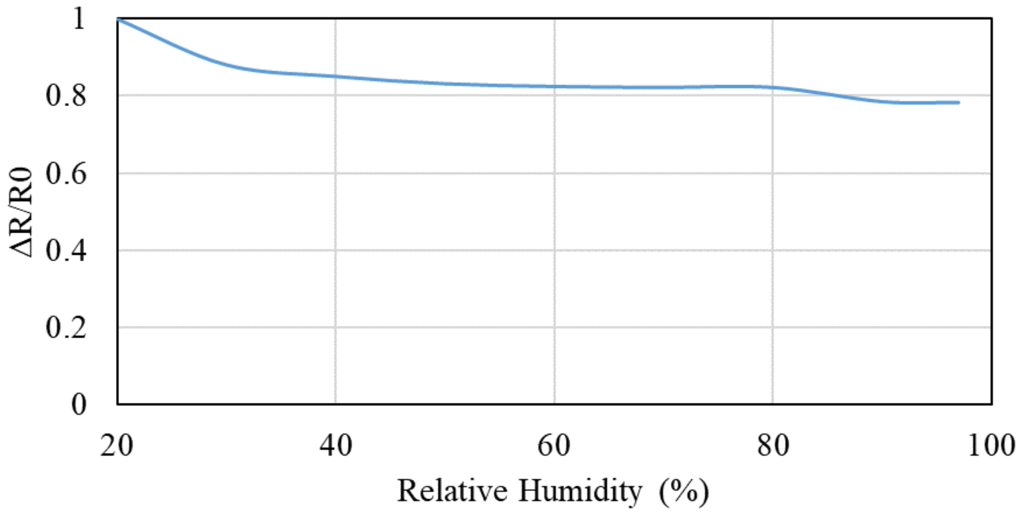

Supplementary Figure 10. the normalised resistance change across the composite for a varying relative humidity, at 20°C.

To further demonstrate the material's thermistor response is almost unaffected by humidity variations, two temperature sweeps were carried out. First, the temperature was swept in dry conditions for a relative humidity under 20%. The same temperature sweep was repeated for a relative humidity exceeding 90%. Supplementary Figure 11 shows the measured thermistor response in both setups, where it can be seen that the temperature-resistance relationship remains mostly unchanged. The maximum observable temperature is in-line with the response in Supplementary Figure 9, for standard room humidity (around 50%).

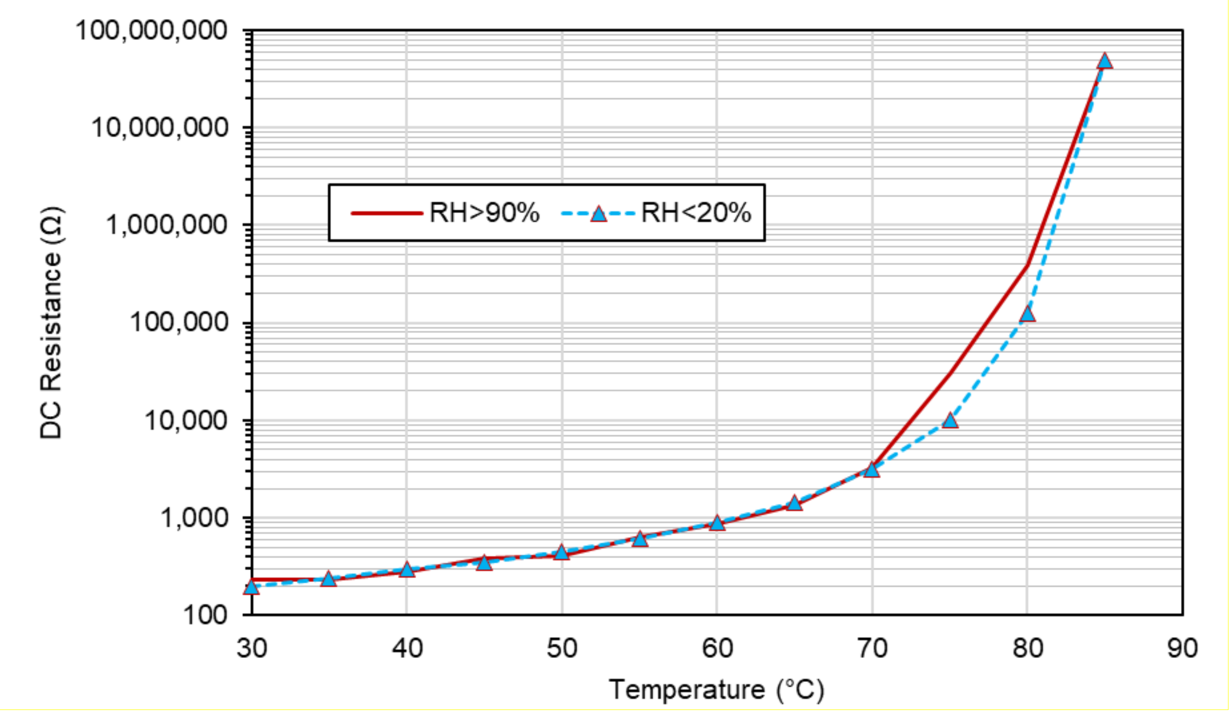

Supplementary Figure 11. The thermistor's DC response in a dry (RH<20%) and humid (RH>90%) environments, showing minimal variations.

Supplementary Note 8: Thermistor's TGA and DSC Response

Both DSC and TGA were used to evaluate the material's response. Supplementary Figure 13 shows the TGA of the material. It can be observed that there's under 0.01% weight change in the material under 200ºC, the sensing range considered throughout the work. Up to 400ºC, over 95% of the material's weight is maintained, which is over 150ºC higher than the maximum temperature sensing range investigated in the work.

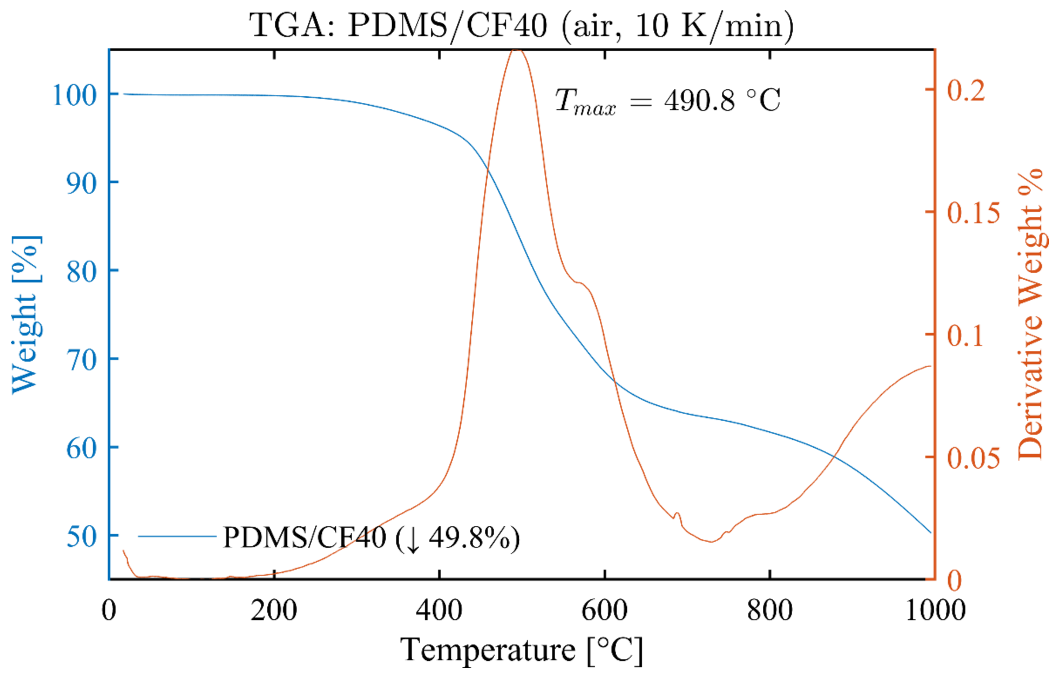

Supplementary Figure 13. Thermogravimetric Analysis of the 40wt.% CF composite.

The material's DSC is shown in Supplementary Figure 14. Over two cycles, the material exhibits a very repeatable response.

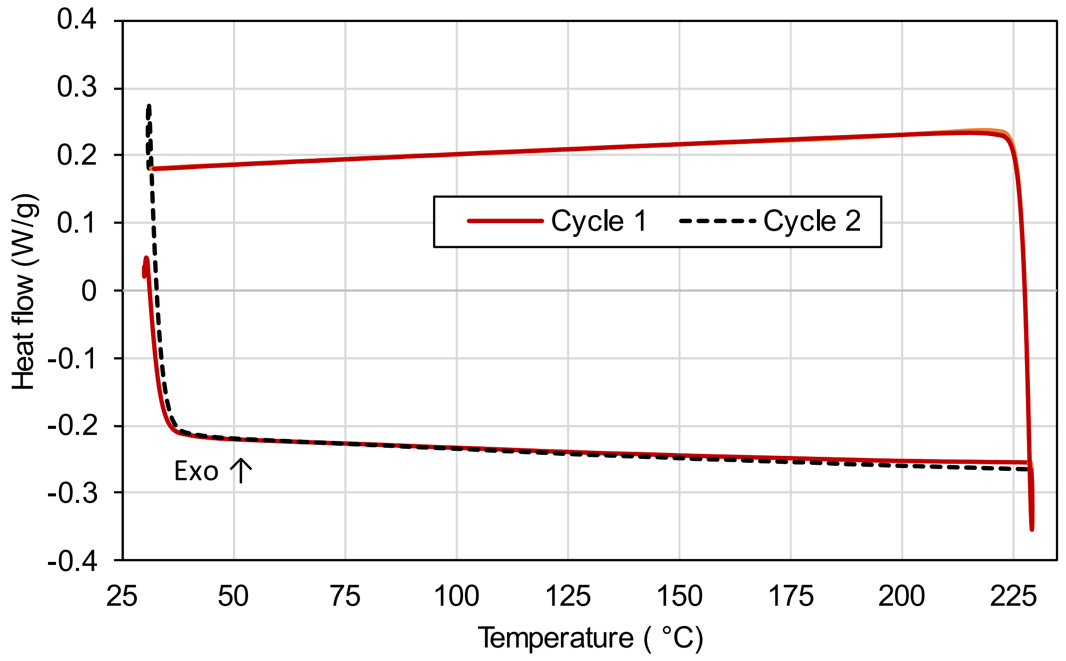

Supplementary Figure 14. DSC thermograph of the 40wt.% CF composite.

Supplementary Note 9: Composite Coefficient of Thermal Expansion

The coefficient of thermal expansion (CTE) of short carbon fibre (SCF) reinforced PDMS composites is calculated here utilising the rule of mixture formula meant for discontinuous nanofiber-reinforced composites appropriately accounting for fibre aspect ratio, orientation, and alignment. Due to the fabrication process, the SCFs become somewhat aligned in the in-plane (XY plane) direction, and therefore, the alignment of SCFs in the in-plane direction leads to an expectation that the CTE in the longitudinal direction, denoted as α_l, will differ from the CTE in the out-of-plane (XZ plane) direction, denoted as α_t. However, we provide an isotropic estimate of the CTE of the PDMS/CF composite through the following equation:

$$\alpha_c = \left(1 - \emptyset_f - \emptyset_p\right) \alpha_m + \emptyset_f \beta_f (1 + \kappa \cos^2 \theta) \alpha_f \quad (1)$$

where, α_m represents the CTE of the PDMS matrix, $\emptyset_f$ stands for the volume fraction of the fibres, $\emptyset_p$ is the volume fraction of the pores (assumed to be 0.02), and α_f denotes the CTE of CF. To account for the anisotropy of the composite, β_f represents the CF's aspect ratio, and κ is the anisotropy ratio.

Note that CF exhibits a negative CTE. The CTE of CF can vary, ranging from -1x10^{-6} /°C to 2.3x10^{-6} /°C, depending on its type and manufacturer. However, for the specific CF being considered here, $\alpha_f = -0.64 \cdot 10^{-6}$ /℃, while for PDMS, $\alpha_m = 300 \cdot 10^{-6}$ /℃.

Supplementary Figure 15 depicts the CTE as a function of the CF weight fraction; the density was experimentally measured for calculating the volume fraction (see the Methods); the densities used are 1.800 g/cm³ and 0.965 g/cm³ for CF and PDMS, respectively. The densities of the composites were measured as 1.16 g/cm³ for PDMS/CF25 and 1.25 g/cm³ for PDMS/CF40, while values for PDMS/CF30 and PDMS/CF40 were interpolated to approximately 1.19 g/cm³ and 1.22 g/cm³, respectively. This allows for the calculation of the volume fraction of the fibres in the composite, denoted as $\varphi_f = w_f * \rho_c/\rho_f$, where w_f is the weight fraction of the fibre, and ρ_c and ρ_f represent the density of the composite and fibre, respectively. The obtained results are in-line with those reported in the literature, for PDMS/CF composites [S2]

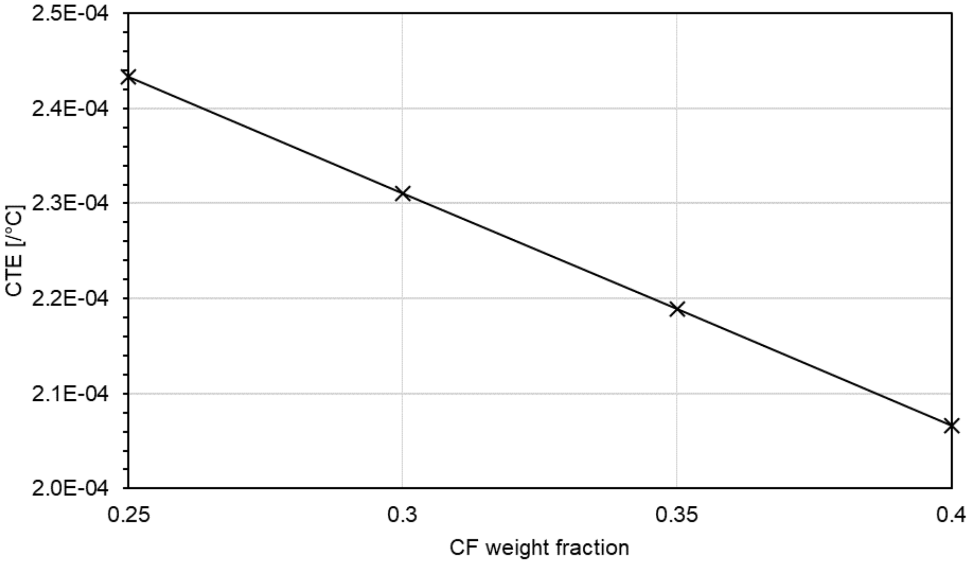

Supplementary Figure 15. CTE of the composite as a function of CF weight fraction for both the in-plane and out-of-plane directions, respectively.

While the changes in the CTEs were not measured as a function of temperature, it is anticipated that within the temperature range examined here, α_c is expected to decrease. This is due to the in-plane α_l being primarily influenced by the fibers. CF exhibits a negative CTE, which decreases as temperature rises, whereas the CTE of the PDMS matrix is expected to increase with temperature. However, it should be noted that within the temperature range considered in this study, these effects can be safely assumed to be negligible. This is also reflected in the RF electrical response, where the observed material response (see Figure 3) is mostly uniform over the tested range of temperatures.

Supplementary Note 10: Temperature Cycling and Thermistor Anisotropy

To test the thermistor's repeatability, it was placed in an environmental chamber (WKL 100) and the temperature was cycled between 20°C and 50°C. The in-plane resistance, XY plane, and the out-of-plane resistance, Z plane, were logged using the Keighley 2001 DMM, to investigate the material's anisotropic TCR. The measured change in resistance is shown in Supplementary Figure 16.

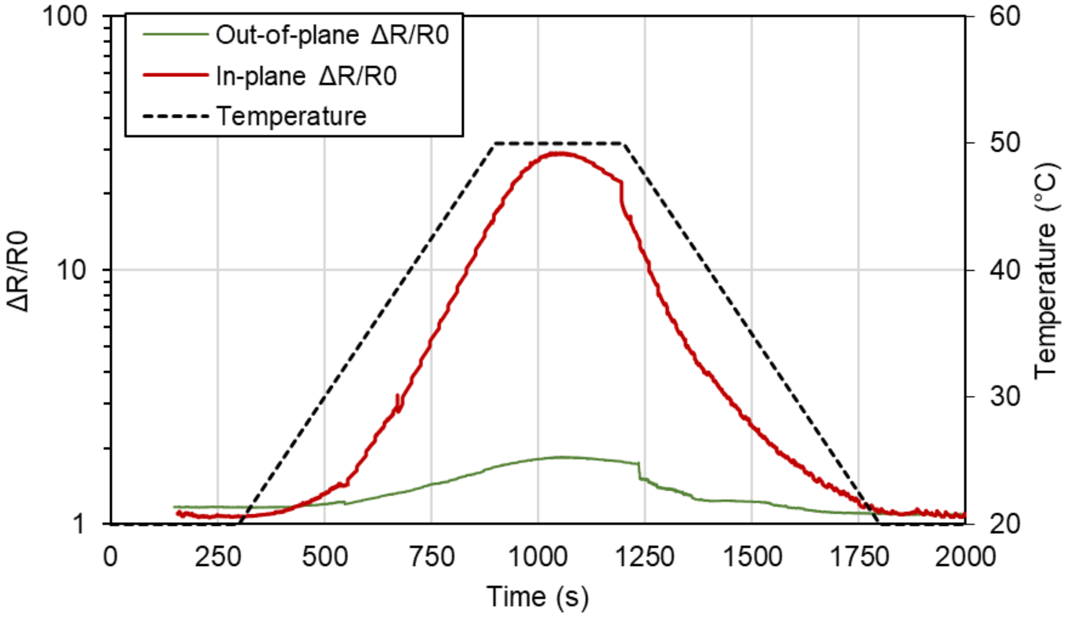

Supplementary Figure 16. normalized resistance change over a temperature profile (shown as a dashed line) for the thermistor in the X direction (in-pane), and the Z direction (out-of-plane).

The temperature profile from Supplementary Figure 16 was cycled 14 times in a period of 30,000 seconds. The resistance changes for the 40% CF composites are shown Supplementary Figures 17. The cyclic response for the other formulations was found to be consistent with the 40% composite.

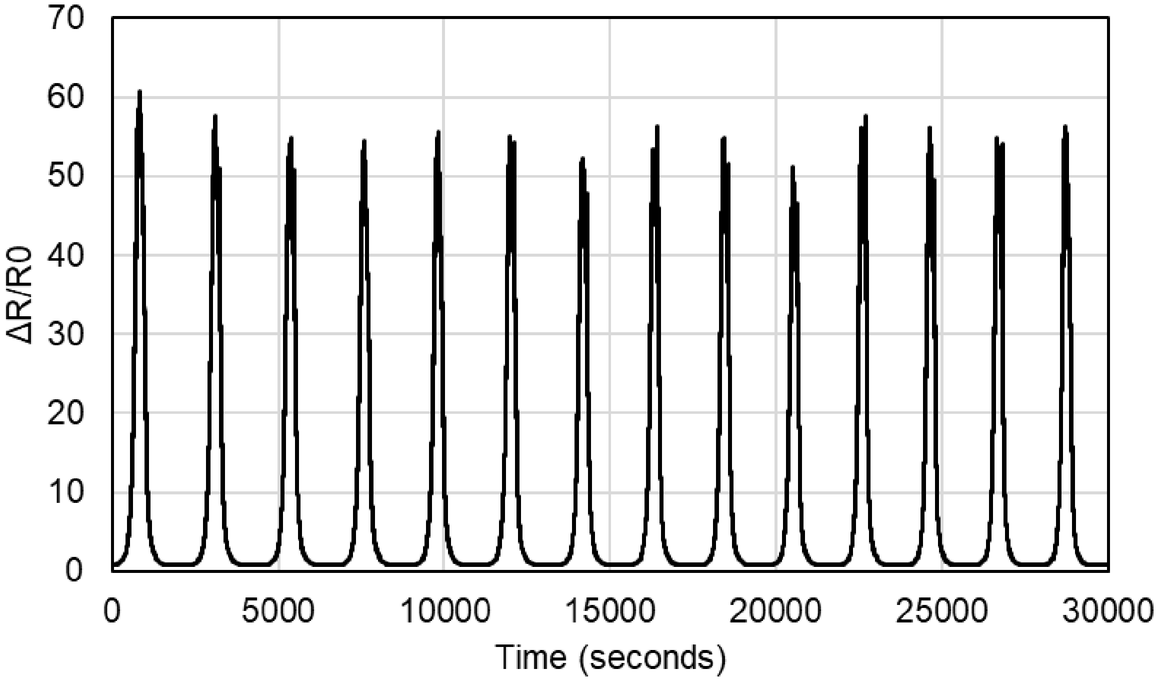

,

Supplementary Figure 17. Normalized cyclic resistance change for the 40% CF/PDMS composite, in the XY plane, between 20℃ and 50℃.

Supplementary Note 11: Broadband RF Permittivity and Loss Extraction from a Transmission Line.

As the composite is used in planar microstrip components (resonators and antennas), the relative permittivity observed by a microstrip line is extracted. A long microstrip line (48 mm) was characterised up to 19.5 GHz to measure the apparent relative permittivity. The permittivity is extracted from the propagation constant of the microstrip line. The VNA's measurement plane was extended to exclude the effect of the SMA to microstrip transition. The effective permittivity of the line is calculated as:

$$\epsilon_{eff.} = \left(\frac{\angle S_{12} + \angle S_{21}}{2(\Delta l \times k_0)}\right)^2 \tag{3}$$

$$k_0 = \frac{2\pi f}{c} \tag{4}$$

where Δl is the length of the line, k_0 is the free-space phase constant, $\angle S_{12}$ and $\angle S_{21}$ are the phase delay of the forward transmission through the line, and c is the speed of the light. The effective permittivity is then used to calculate the permittivity of the substrate, based on the dimensions of the microstrip line. The loss tangent $tan\delta$ of the material was calculated using the measured attenuation of the line, and is given by:

$$\alpha_d = \frac{k_0\epsilon_r(\epsilon_{eff} - 1)\tan\delta}{2\sqrt{\epsilon_{eff}}(\epsilon_r - 1)} \tag{5}$$

the bulk conductivity was then calculated using

$$\sigma = \tan\delta\, \epsilon_r\epsilon_0\omega. \tag{6}$$

The sheet resistance of the material, which can be used to simplify the frequency-domain modelling, is obtained using:

$$R_{sheet} = \frac{1}{\sigma t}, \tag{7}$$

where t is the thickness of the substrate.

Supplementary Figure 18 shows the extracted permittivity over frequency. The line was placed on the hotplate in the same setup used to characterise the resonators, and its transmission response was measured at room temperature (around 22°C) and when heated to 130°C; the temperature of the composite was verified using an infrared camera. The observed permittivity response is in line with that observed in Figure 2(i), where the composite is most sensitive to temperature up to 5 GHz. Thus, all the sensing resonators, antennas, and RFID tags are designed for operation under 4 GHz. The extracted sheet resistance of the substrate up to 19.5 GHz is shown in Supplementary Figure 19.

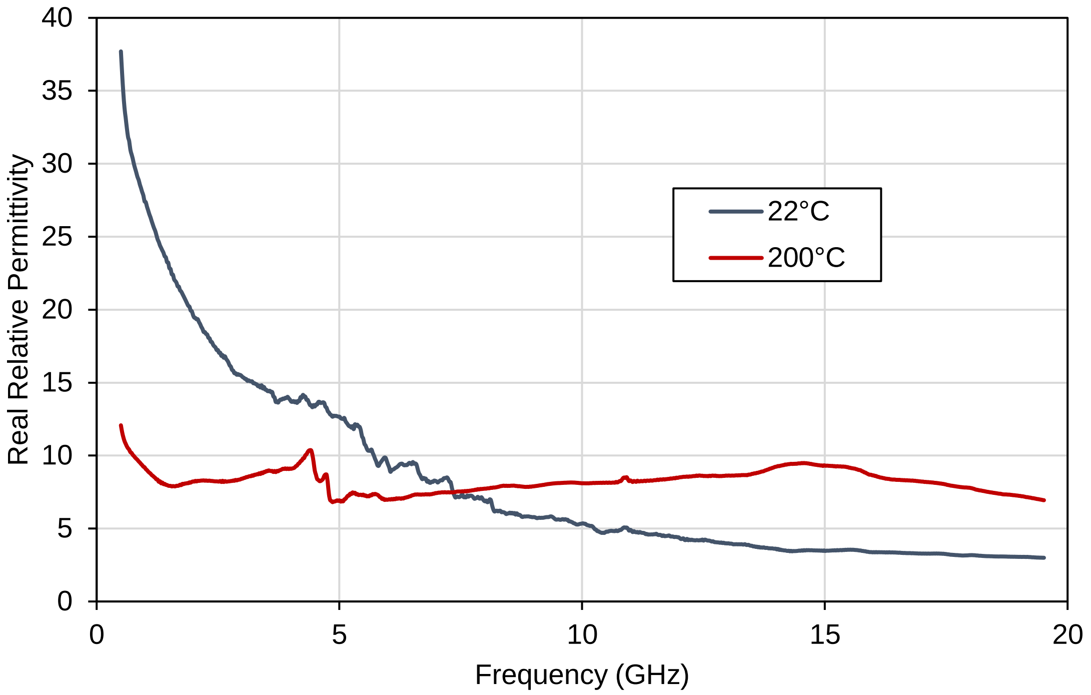

Supplementary Figure 18. Extracted real permittivity of the composite using a microstrip line's transmission line response up to 19.5 GHz.

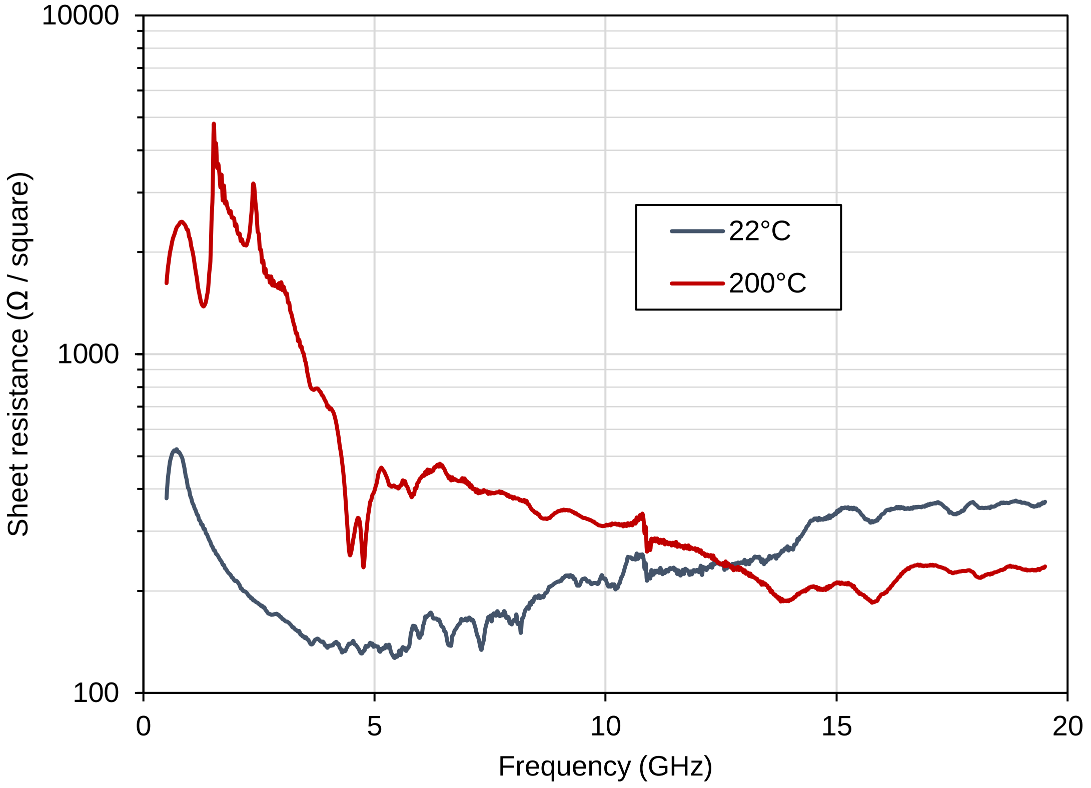

Supplementary Figure 19. Extracted sheet resistance of the composite from the insertion losses of the transmission line, in the XY plane (in-plane) up to 19.5 GHz.

From the 0.5-20 GHz measurements, it can be seen that the material's highest sensitivity to temperature is under 5 GHz. This is attributed to the increased dielectric loss in the substrate at higher frequencies. As all sensing resonators are designed for operation under 4 GHz, the material's broadband relative permittivity and conductivity were measured using a microstrip line up to 6 GHz. The lower measurement frequency was extended to 10 MHz, to approach the low-frequency response of the material; this is limited by the VNA's calibration, to ensure a maximum to minimum frequency ration under 1000:1.

The temperature of the hot plate (setup shown in Supplementary Figure 29) was swept and the temperature over the surface of the thermistor was verified using an infrared camera. The permittivity and conductivity were extracted using supplementary equations (4) to (7). Supplementary Figure 20 shows the measured real relative permittivity of the material as well as the conductivity, extracted from the tanδ of the substrate. The logarithmic axes are used to improve the visualisation of the data's trend, due to the non-linear response; the article's full dataset shows the exact data.

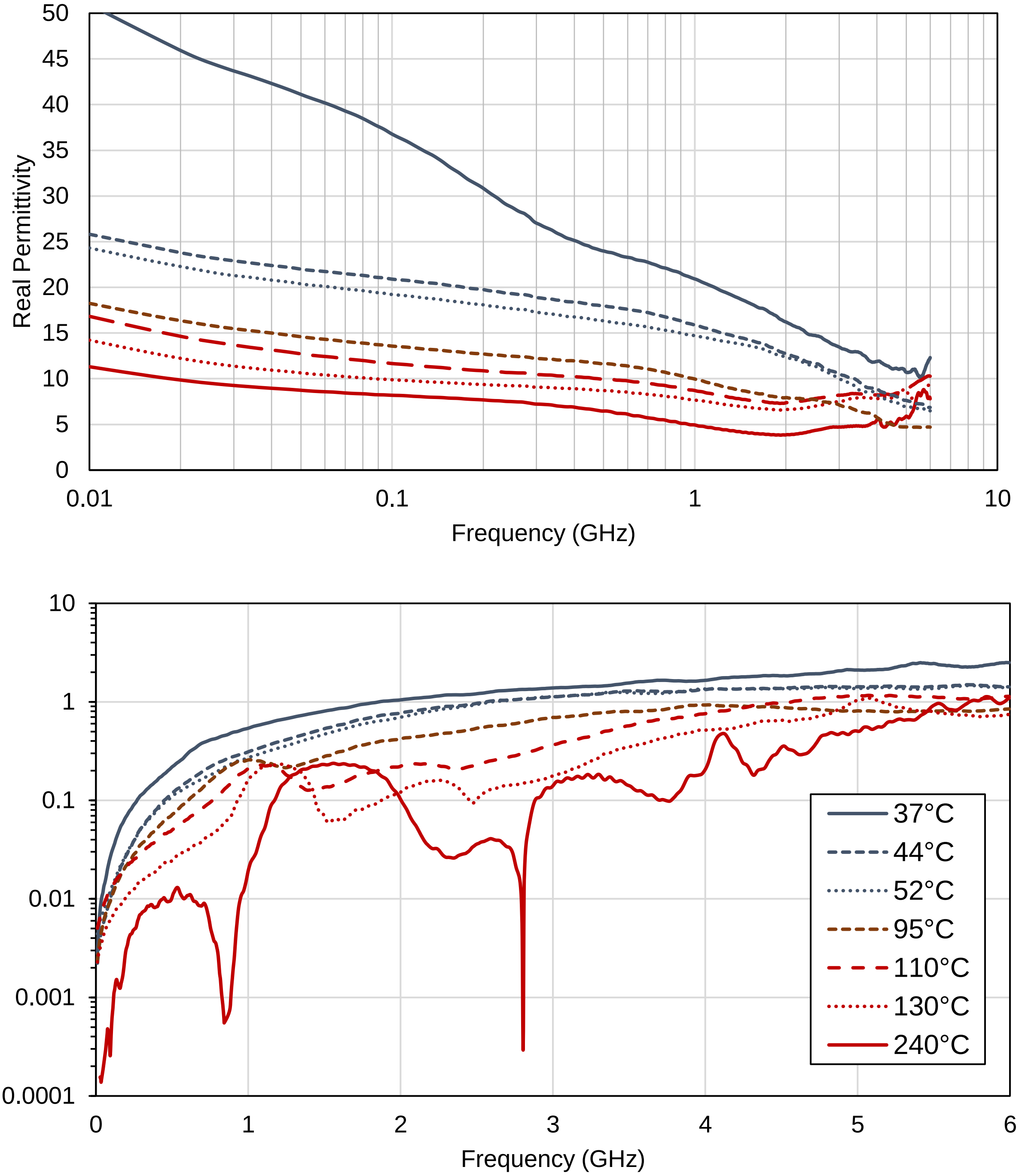

/

Supplementary Figure 20. Measured RF properties of the material observed through a 56 mm-long microstrip line.

Supplementary Note 12: Band-Specific Non-Contact (Waveguide) RF Conductivity Measurement.

Augmenting the microstrip results, a non-contact approach was used inside a waveguide, for extracting the material properties at higher frequencies using the Nicholson-Ross Weir (NRW) method. Broadband methods have reduced accuracy for lossy or high permittivity material. Therefore, the intrinsic conductivity and permittivity of the material were re-measured using the NRW method in a waveguide.

Supplementary Figure 21 shows the measurement setup for the composite inside the waveguide. As seen in Supplementary Figure 21(c), the thermistor was fitted to the waveguide's dimensions to eliminate any air gap. Given the electric field distribution in the waveguide, the measured conductivity represents an average of the high- and low-conductivity regions in the composite. Therefore, it is expected to be higher than the DC conductivity, which can be significantly reduced by contact resistance between the CFs.

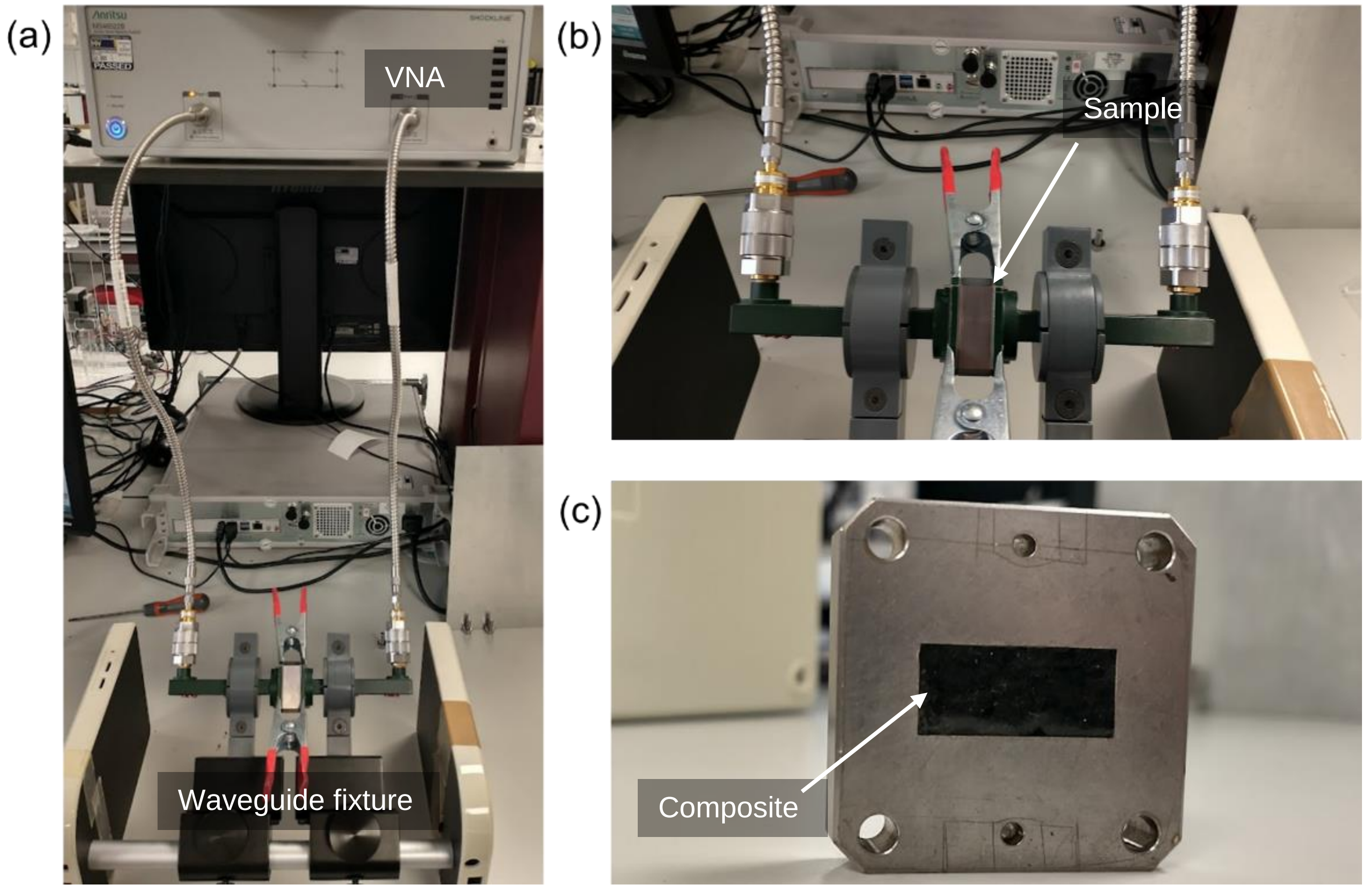

Supplementary Figure 21: (a) waveguide measurement setup; (b) close-up picture showing the "through" section in which the composite sample is inserter; (c) the composite filling the waveguide's cavity.

Supplementary Figures 22 shows the broadband measured conductivity of the composite, both in-plane (Y direction) and out-of-plane (Z direction). The out-of-plane conductivity was measured by stacking sliced samples of the composite inside the waveguide, with no air gaps between the composite slices. As the electric field inside the waveguide interacts with an average of the material, the contact resistance between the sheets would have a negligible effect on the measured conductivity of the sample.

As seen in Supplementary Figure 22, the conductivity is both significantly lower, out-of-plane, and also less dependent on the ratio of the CFs in the material. This is line with the DC response of the material, and the assumption that the CFs align in the direction of casting, i.e. the XY plane.

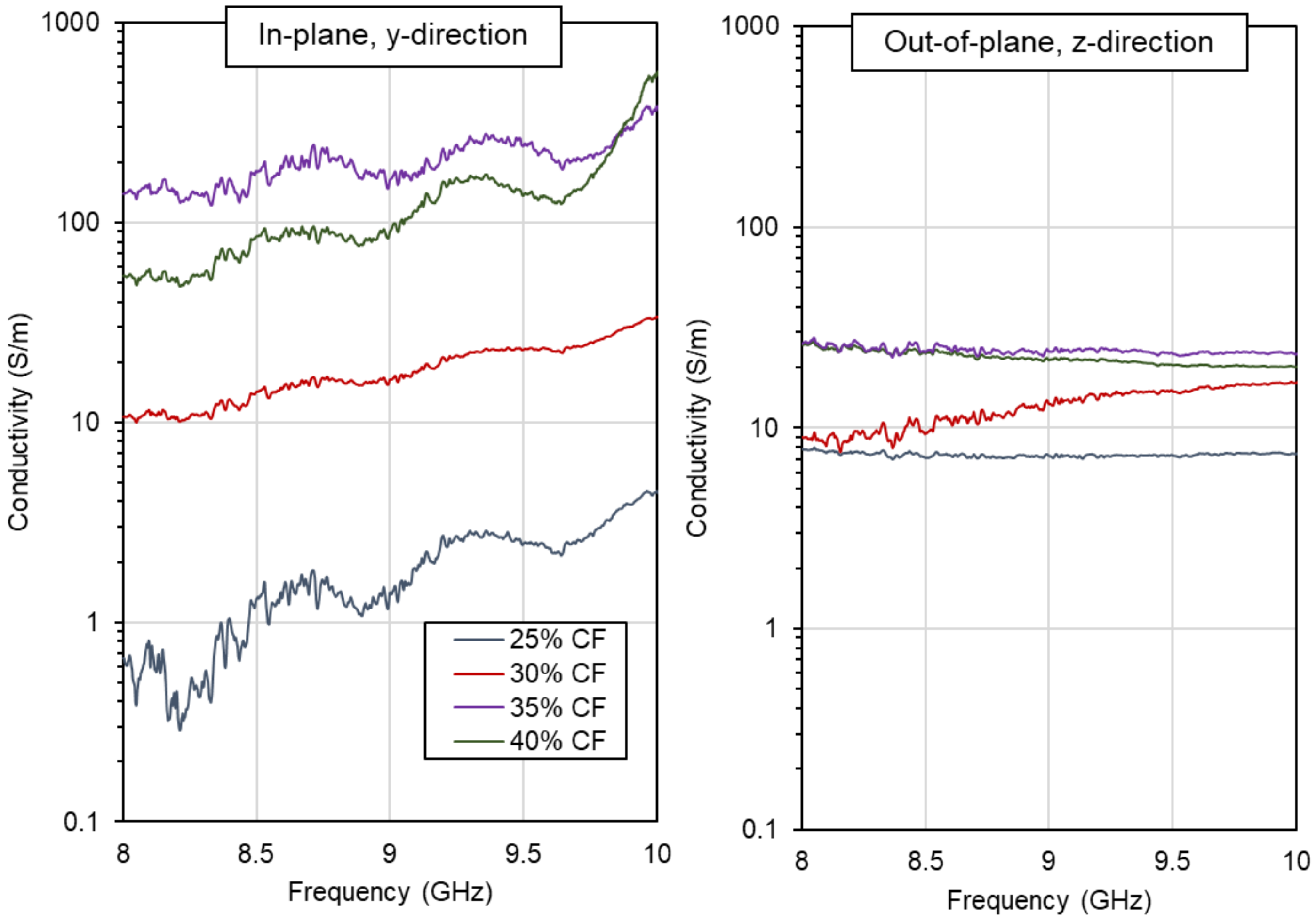

Supplementary Figure 22. Measured RF conductivity using the NRW method in a WR-90 waveguide.

A key difference between the conductivity measurement at RF using the waveguide setup, comparted to DC conductivity measurements, is the dependence on the sample's thickness. Therefore, it is expected that the observed bulk resistivity, and consequently sheet resistance, will be lower than their DC counterpart. To demonstrate this experimentally, two composite samples are placed back-to-back inside the waveguide. The two samples were separated by a <0.1 mm-thick low-loss polymer film. Supplementary Figure 23 shows the measured sheet resistance and apparent bulk conductivity, for the individual composites and when both are measured collectively. Therefore, it can be seen that the measured RF conductivity will be dependent on the material's thickness.

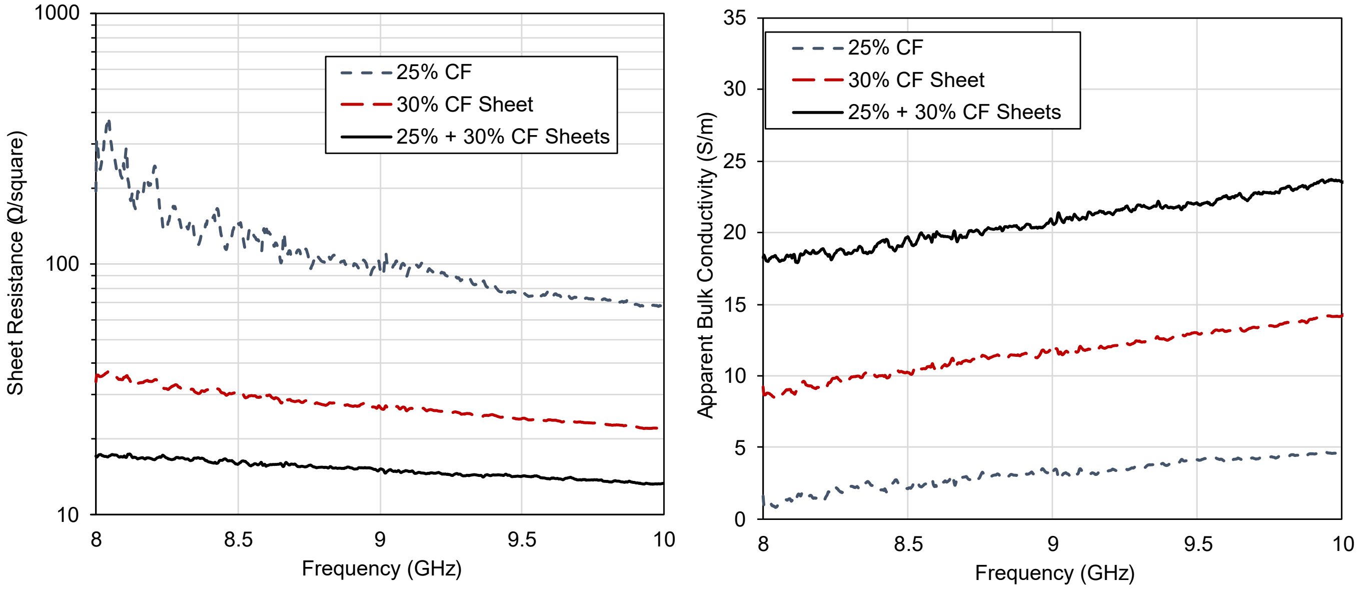

Supplementary Figure 23. Measured RF sheet resistance and conductivity for two samples placed back-to-back, with an insulating sheet for separation, inside the waveguide.

To explore the material's response at higher frequencies, a higher frequency waveguide (WR42) was used to measure the material's permittivity and loss tangent from 18 to 26 GHz. The waveguide measurements was used as the microstrip measurements were only possible up to 20 GHz, due to the difficulty in measuring the microstrip accurately above 20 GHz (given the SMA connector's limit). Due to the difficulty of heating the material inside the waveguide, the material and enclosing waveguide section were pre-heated for over 10 minutes before placing the sample within the measurement fixture. The pre- and post-heating response is shown in Supplementary Figure 24.

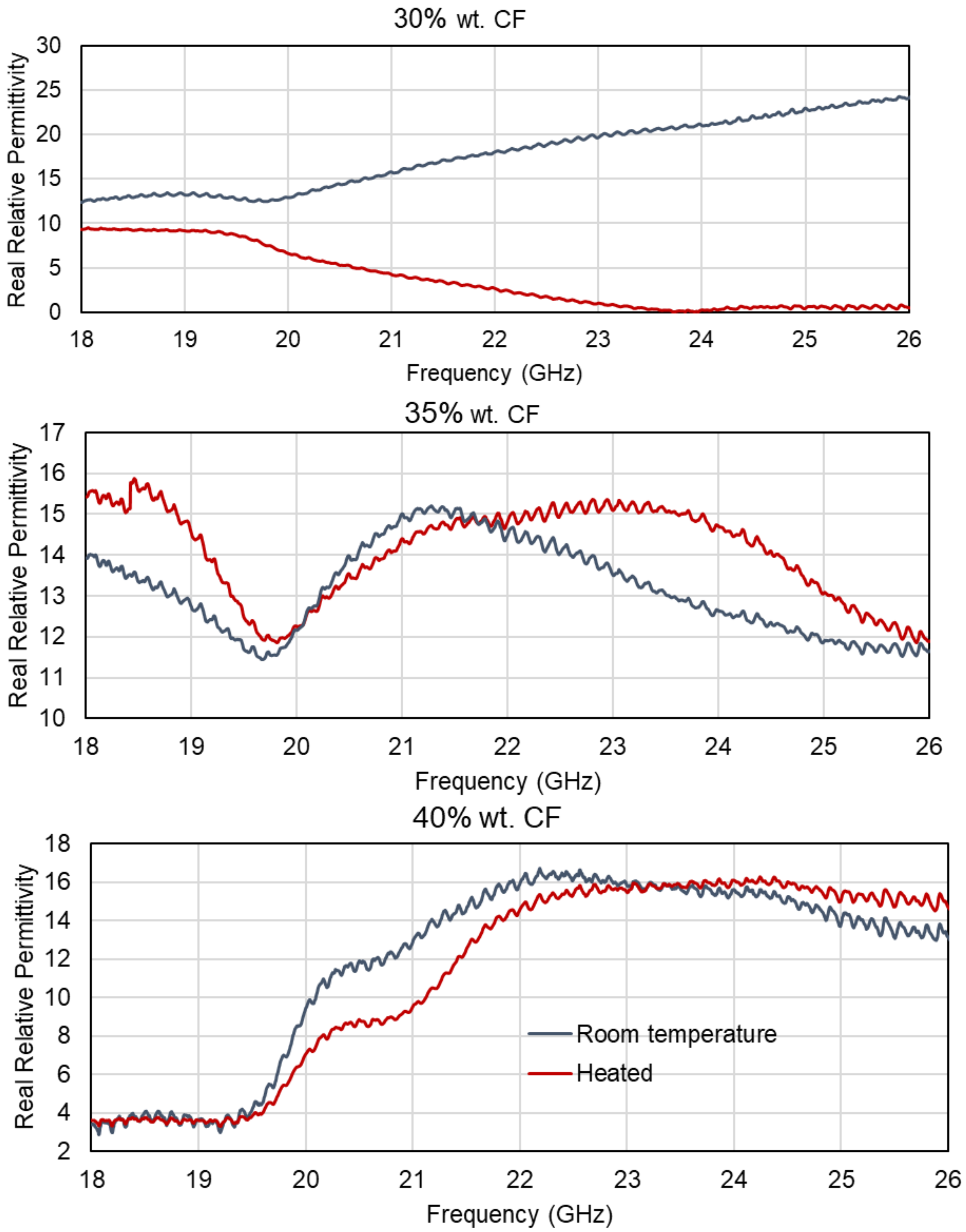

Supplementary Figure 24. measured real permittivity of the sample in a WR-42, for a heated and room temperature sample.

To cross-validate the waveguide measurements, the measured permittivity of the 40 wt.% CF composite was compared to the values extracted from the transmission response of the microstrip line. As seen in Supplementary Figure 25, the extracted permittivity from the two different setups, and using two different material batches, gives a similar relative permittivity response, in the overlapping frequency range (18 to 19.5 GHz). The NRW-extracted permittivity from the waveguide measurements exhibit limited oscillations, which are due to the standing waves in the waveguide, and due to variations in the sample's thickness and the roughness of its edges.

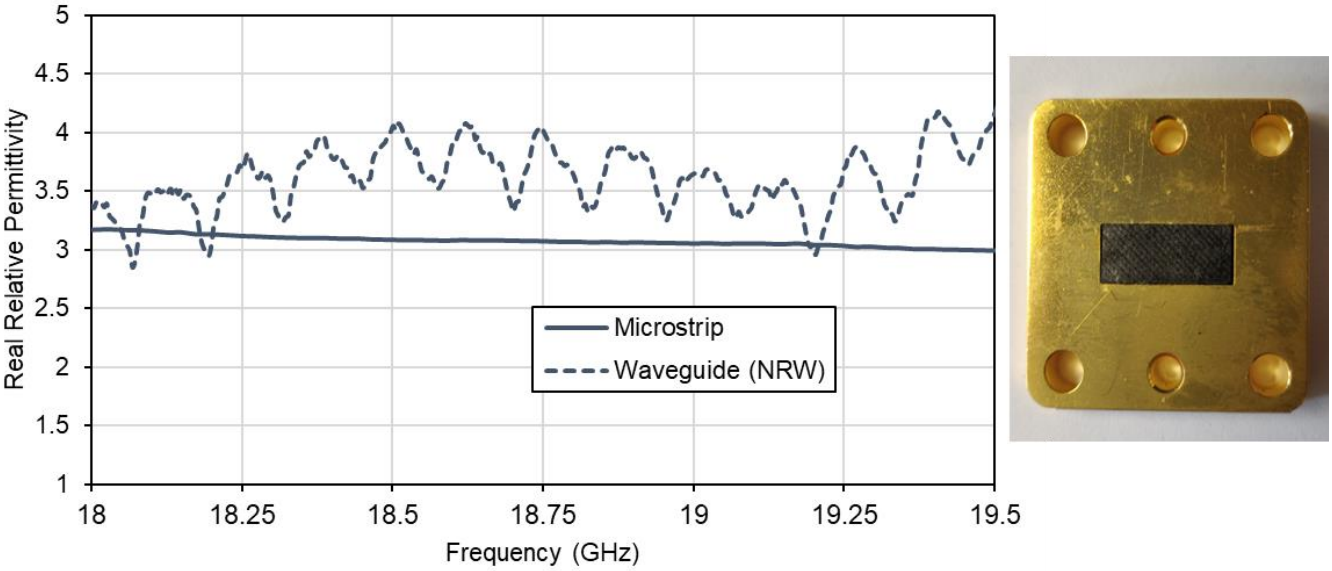

Supplementary Figure 25. Comparison of the measured permittivity from the microstrip method and the free-space waveguide measurement in the WR42 waveguide; the photograph shows the material inside the WR42 waveguide section, used to heat the sample.

Supplementary Note 13: RF Anisotropy's Impact on the In-Plane Material Properties

Due to the material's anisotropy, it is key to demonstrate that the sensor's performance is not dependent on the alignment of the resonator's geometry with the CFs. Two microstrip lines were measured across the same thermistor substrate in orthogonal directions, as illustrated in Supplementary Figure 26. The measured transmission response was used to extract the apparent permittivity and conductivity, using the approach detailed in Supplementary Note 12. It can be seen that the room-temperature response in both directions is highly comparable.

As for the temperature-dependent response, it can be seen that a high sensitivity is achieved in both directions, in Supplementary Figure 27. The variation observed between the X and Y plane is attributed to the aspect ratio of the CFs (around 13.7:1). Therefore, while the material is highly anisotropic in the out-of-plane orthogonal planes (e.g. XZ), the in-plane RF response of the material is mostly consistent, allowing the placement of the microstrip components to be arbitrary, in the XY plane. However, to achieve the highest sensitivity, the alignment of the microstrip structure, with respect to the fibres, needs to be considered.

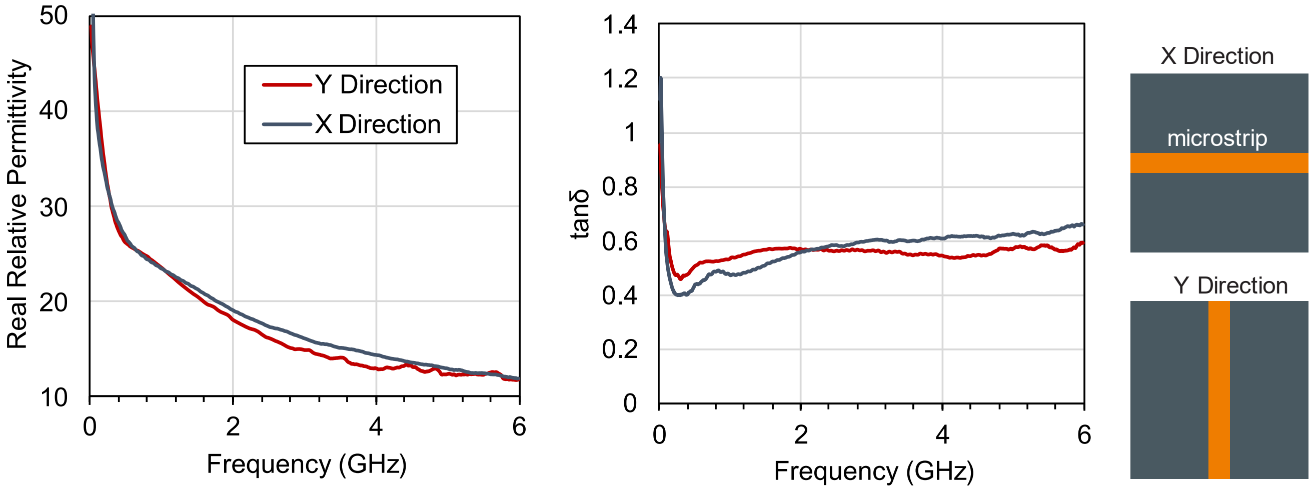

Supplementary Figure 26. measured relative permittivity of the composite as observed by a microstrip line, in both the X and Y directions.

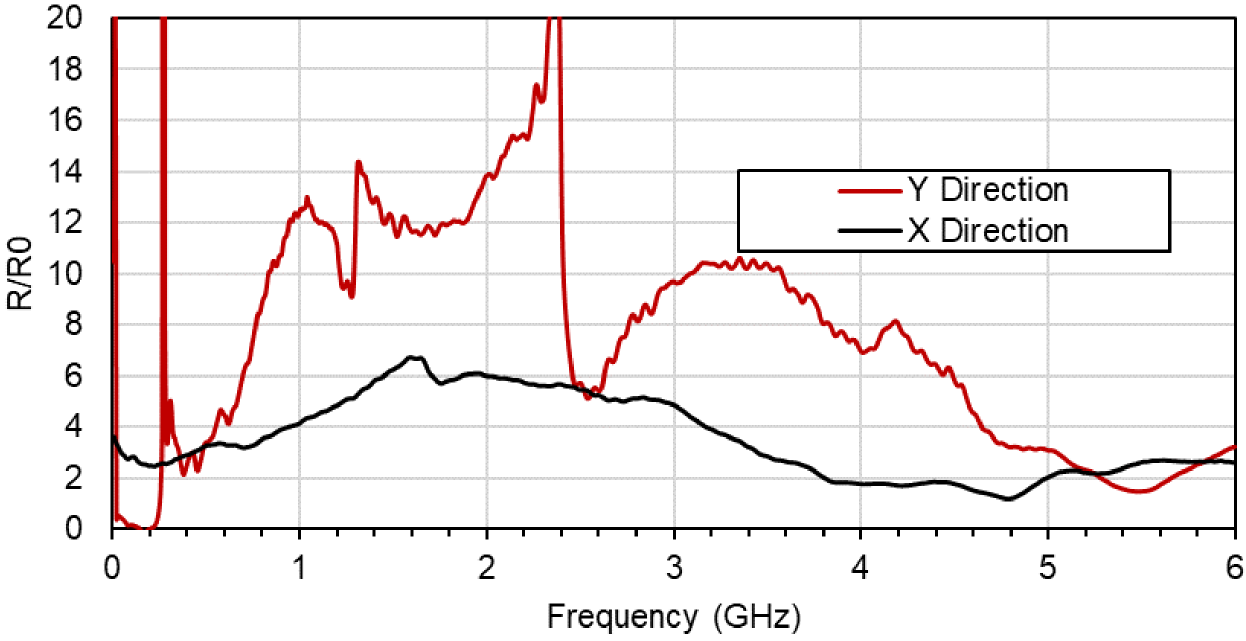

Supplementary Figure 27. measured change in the resistivity of the composite as observed by a microstrip line, in both the X and Y directions.

Supplementary Note 14: Flexible and Printable Temperature Sensing Materials Comparison.

Supplementary Table 2. Comparison with previous solution-processed temperature sensors.

**Reference**	Device	Material	Temperature Range (°C)	TCR (°C^{-1})
**[S3]**	Transistor	Graphite/ alumina	20-100	4.4%
**[S4]**	Thermistor	Molybdenum Diselenide (MoS_2)	0-100	1.75%
**[S5]**	Thermistor	Graphene hydrogels	26-101	2.04%
**[S6]**	Thermistor	Graphite-PDMS	20-80	5.73%
**[S7]**	Thermistor	PEDOT:PSS/PET/PI	25-55	1.79%
**[S8]**	Thermistor	$BaTiO_3$/PET	26-55	4.19%
**[S9]**	Transistor	Ni/PET	25-70	9.20%
**[S10]**	Transistor	Silver PET	17-36	91.0%
**[S11]**	PTC Thermistor	Carbon nanotubes	35-75	17.3
**[S12]**	PTC Thermistor	PEDOT:PESS-CNT	10-50	31.0
**[S13]**	PTC Thermistor	Graphite-copolymers	30-34.5	100,000
**[S14]**	NTC Thermistor	PTF/Mxene/Fe	20-80	1.32%
**[S15]**	NTC Thermistor	PTF/Mxene	0-80	5.27%
**This work**	PTC Thermistor	CF/PDMS	25-50 (DC); 25-205 (RF)	200 to 2,680

From Supplementary Table 2, it can be seen that the proposed thermistor uses an inexpensive conductor and binder, both of which have demonstrated biocompatibility [S16, S17]. Furthermore, as shown in the later sections for the microwave resonators, the proposed composite can be moulded into different geometries, enabling large-area electromagnetic structures to be designed.

The observed variations in the frequency sensitivity may be attributed to differences in the CF concentration around the composite, which changes the permittivity change observed by the highest *E*-fields.

Cost and large-scale applications:

From a cost and scalability perspective, the highest sensitivity materials in Supplementary Table 2 rely on nanoparticles which are substantially higher in cost, or unavailable commercially, compared to the chosen CFs. To explain:

- The CF used in this work (P-MCF-004 Easy Composite) cost between £100/kg and under £20/kg from easycomposites.co.uk.
- Commercially-available carbon nanotubes (CNTs) are typically in excess of £30,000/kg (based on item number 659258 from Sigmaldrich.com).

Thus, thermistors formulated based on such high-cost nano-scale materials will inherently be limited to very small devices which cannot be practically scaled up to the composite sizes demonstrated in this work. As a result, their adoption in RF sensing and with intrinsically-wireless readout will require complex geometries and high-sensitivity instrumentation to resolve their low sensitivity. This is further evidenced by the low sensitivity (in MHz/^{o}C) of reported RF temperature sensors [S16-S25], which mostly avoid such high-cost nanomaterials due to their limited areal scalability. [S16-S25] are compared in Supplementary Table 3.

Supplementary Note 15: Microwave Resonator 1 Dimensions and Broadband Response.

Supplementary Figure 28(a) shows the dimensions of the first resonator (Resonator 1). The dimensions of resonator 1 were based on the permittivity of PDMS and is therefore mismatched to the 50 Ohm coaxial interface. Thus, the sensory response of this resonator is only used to observe the resonance shifts and their S21 magnitude; later, a second resonator (Resonator 2) was implemented to observe the sensitivity at the structure's first-order resonance. The frequency-domain response of the resonator around its first-order resonance is show in Supplementary Figure 28(b), where the frequency shift and the Q-factor improvement can be clearly observed. Supplementary Figure 29 shows the resonator and the setup.

These results are summarised, over frequency, in Supplementary Figure 30, where a sensitivity of approximately 2 MHz/ºC is observed. Despite the impedance mismatch at the input of the resonator and its apparent simplicity, this would still be considered a significant improvement over state-of-the-art frequency-domain temperature sensors, as compared in Supplementary Note 10.

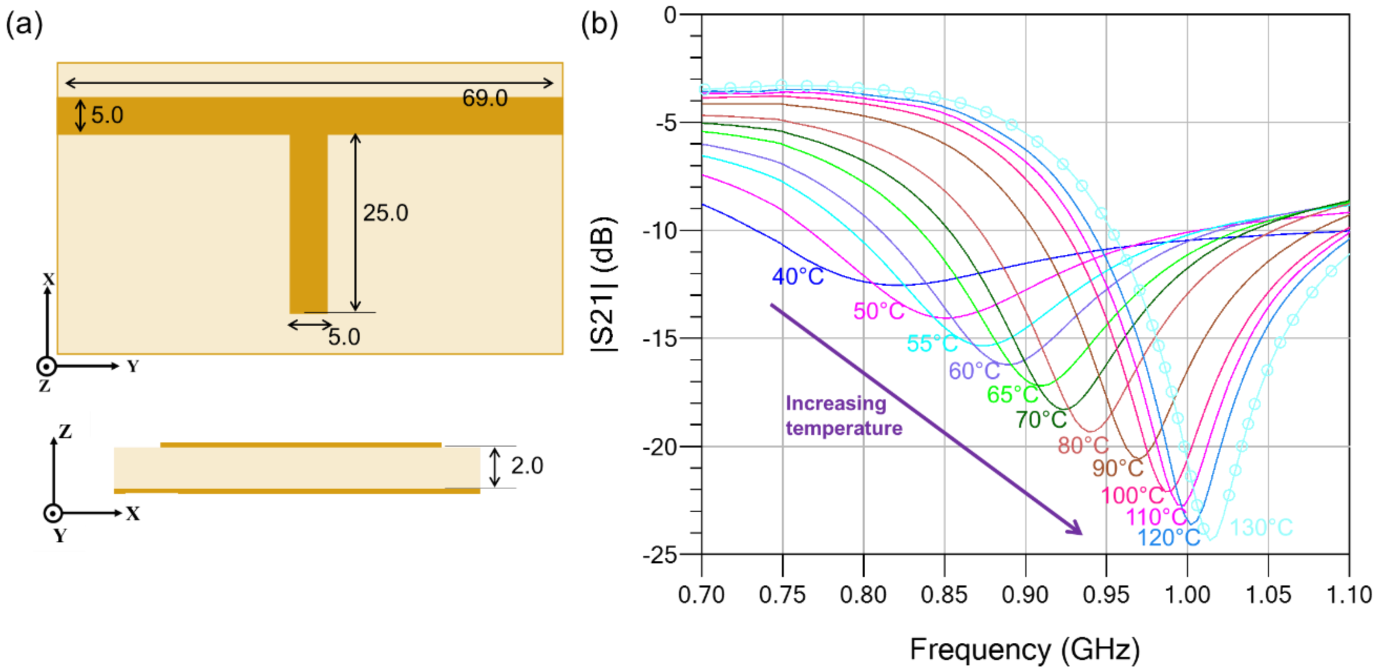

Supplementary Figure 28. (a) Layout and dimensions of Resonator 1, the first T-resonator design used to observe the resonant and non-resonant S21 response over the PMC thermistor substrate. (b) the measured frequency-domain response around F_1.

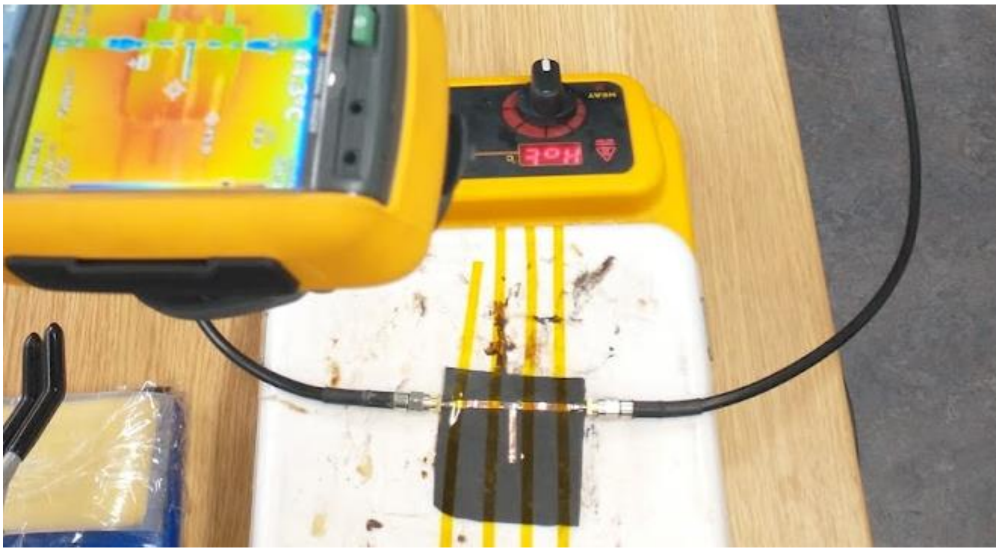

Supplementary Figure 29. Measurement setup of the thermistor-based resonator on the hotplate, with the coaxial cables connected to the VNA; the photograph shows Resonator 2.

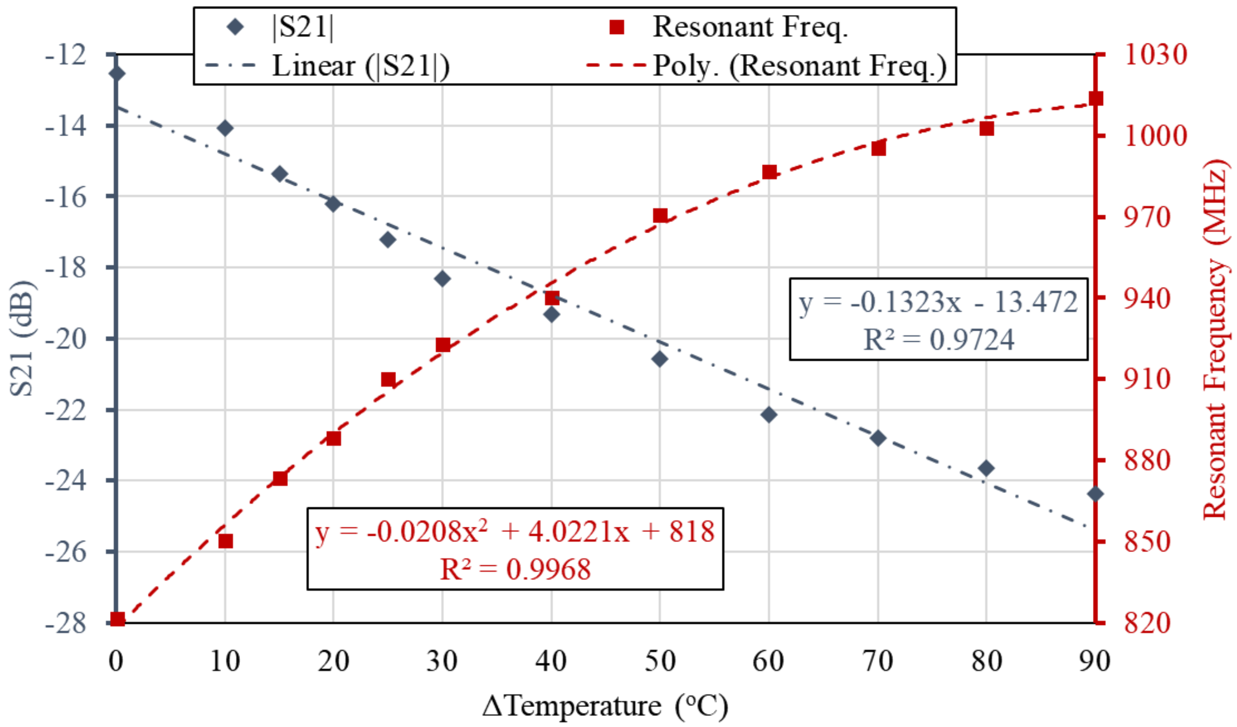

Supplementary Figure 30. Measured transmission-mode resonant sensing using Resonator 1's first-order resonance, through both the frequency and the S11 magnitude. The curve-fitting relations are shown on the plot.

In Supplementary Figure 31(a), the reduced attenuation in the transmission line can be observed, which is attributed to the increase in the in-plane resistance with increasing temperature. In Fig. 31(b), the real relative permittivity shift is observed through the phase change.

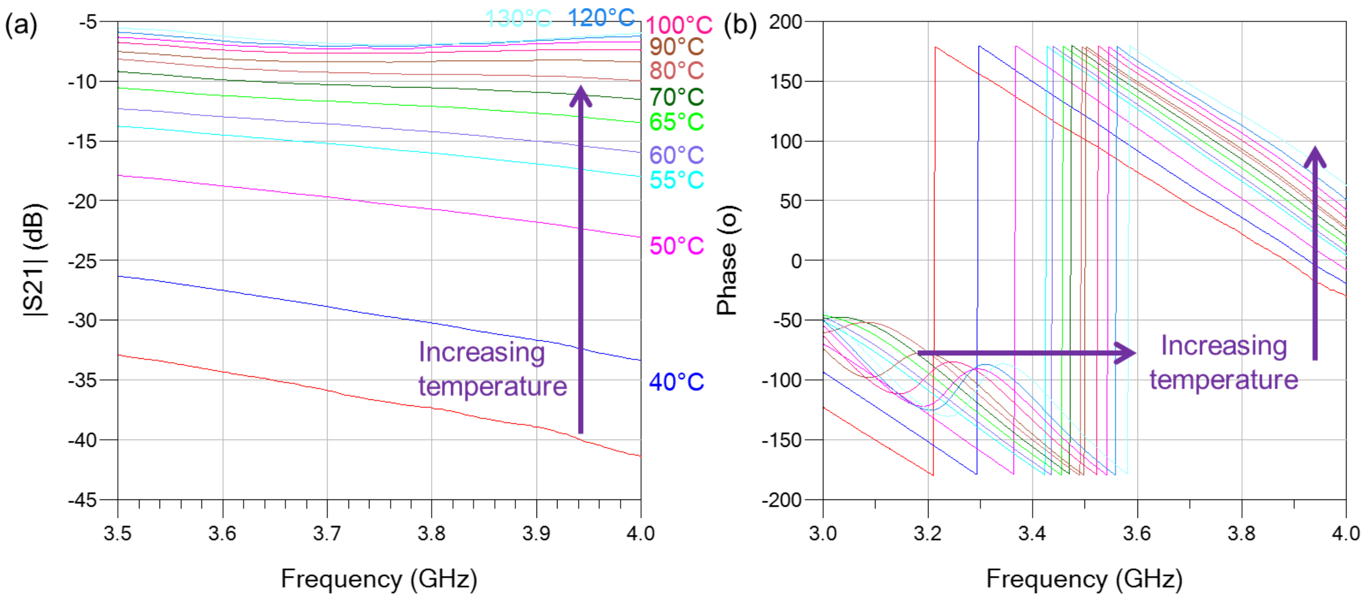

Supplementary Figure 31. The measured magnitude (a) and phase (b) response of Resonator 1 over its non-resonant transmission range, for varying temperatures.

Supplementary Note 16: Resonator 2 Design and Dimensions

From the observed response of Resonator 1, the apparent relative permittivity, inclusive of height non-uniformities and gaps between the copper and the substrate, is around 15 at room temperature. Therefore, Resonator 2 was designed based on narrower traces, to increase the characteristic impedance of the line, reducing the mismatch between the 50Ω coaxial connectors and the microstrip line. At a width of 2 mm and 2 mm substrate height, the characteristic impedance of the microstrip line can be estimated around 40Ω. The dimensions of Resonator 2 are shown in Supplementary Figure 32.

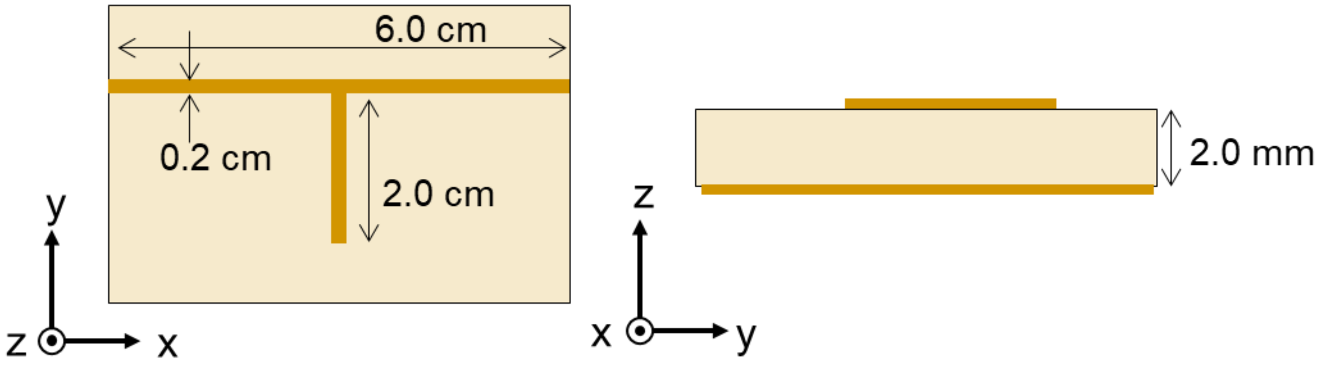

Supplementary Figure 32. Layout and dimensions of Resonator 2.

Supplementary Note 17: Comparison with Previous RF Temperature Sensors

The references depicted in Figure 3(g) are compared in Supplementary Table 3. The temperature sensing range reported in the table represents the full temperature range over which the resonant frequency was observed in the respective work.

Supplementary Table 3. Comparison with previous RF temperature sensors.

Reference	Sensor topology	Temperature Sensing Range (°C)	Unloaded resonant frequency (MHz)	Sensitivity (MHz/°C)	Frequency-Normalized Sensitivity (%)
**[S18]**	Dielectric resonator (chipless tag)	20-370	3,000	0.307	0.0102
**[S19]**	Chipless SRR*	28-1,100	2,420	0.0956	0.00395
**[S20]**	Near-field LC resonator	19-900	33	0.00522	0.0158
**[S21]**	Dual-SRR	23-200	11,930	0.462	0.00387
**[S22]**	Patch antenna	33-77	5,000	0.205	0.0041
**[S23]**	Microstrip resonator	30-80	2,400	0.500	0.0208
**[S24]**	BAW resonator	10-80	2,480	0.100	0.00403
**[S25]**	LC resonator	500-1,200	54.5	0.00357	0.00655
**[S26]**	Cylindrical antenna/ resonator	0-400	10,500	0.35	0.00333
**[S27]**	SAW resonator	-40-120	404	0.0313	0.00774
**This work**	Patch antenna	35-205	2,000	3.17	0.124
	Resonator 2	35-205	1,770	3.29	0.186
	Resonator 1	35-110	820	2.14	0.262

*SRR: split-ring resonator; BAW: Bulk acoustic wave; SAW: Surface acoustic wave

Most of the sensors [S17-S26] are based on standard rigid PCB materials with subtractive fabrication, and are limited to the dimensions of commercially available PCB laminates. Therefore, they are less suited to conformable sensing applications such as on-body operation. It is noted that the maximum temperature sensing range for the proposed samples was limited by the temperature at which the coaxial RF interconnects would get damaged, rather than the frequency at which the material no longer exhibits temperature sensitivity. Previously reported PDMS-based composites have been shown to operate up to 500°C [S27], implying that the proposed composite could potentially be applied in chipless temperature sensing applications beyond 200°C, subject to employing suitable adhesive and conductive traces, and contactless readout. This is also in-line with our experimental TGA results, which show that the material’s mass remains almost unchanged, up to 400 °C**.**

Supplementary Note 18: Patch Antenna Dimensions and Near-Field Characterisation

The layout and dimensions of the patch antenna are shown in Supplementary Figure 33. The width of the feeding line and the substrate's thickness were chosen to match those of the two resonators.

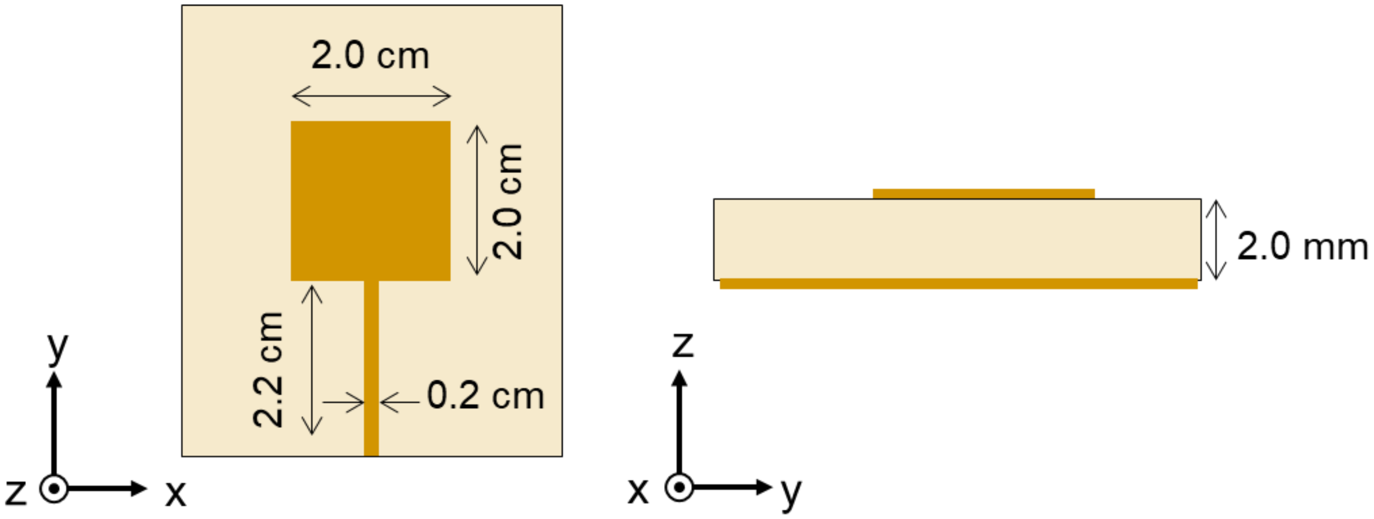

Supplementary Figure 33. Layout and dimensions of the thermistor-based patch antenna, used in the wireless readout (Section 4).

The measurement setup of the near-field gain changes is shown in Supplementary Figure 34. To eliminate any multi-path effects and also minimise the angular dependence, the gain changes were first observed in the near field. The antenna was placed on a hot plate with the near-field monitored using the *E*-field probe (RS E 02, R&S® HZ) over the antenna's broadside direction. The thermal camera shown in the setup was used to ensure the temperature rise over the surface of the composite was uniform.

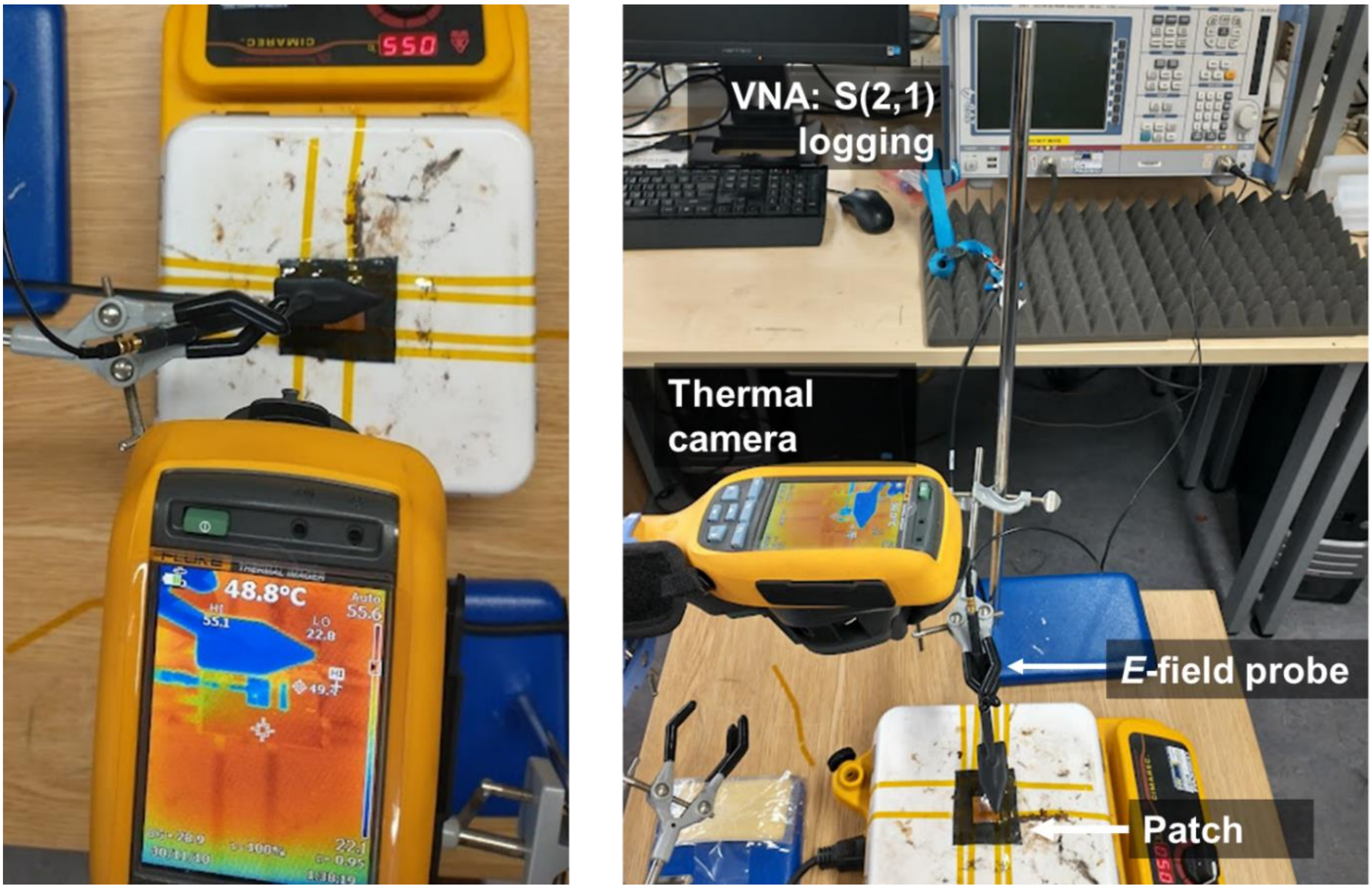

Supplementary Figure 34. Measurement setup of the patch's near-field as a function of temperature, using a VNA and a near-field *E*-probe.

Supplementary Figure 35 shows an infrared photograph of the patch over the hot plate, close to the maximum temperature beyond 200ºC; it is noted that the surface of the composite will be at a lower temperature than the bottom layer, due to cooling from the room's temperature. For instance, the infrared picture shows the hotplate's surface at a maximum temperature of 221.0ºC, with the surface of the composite at a lower temperature of 183.5ºC. Therefore, all measurements primarily reflect the temperature changes, as opposed to the absolute value, which has been verified through the infrared camera.

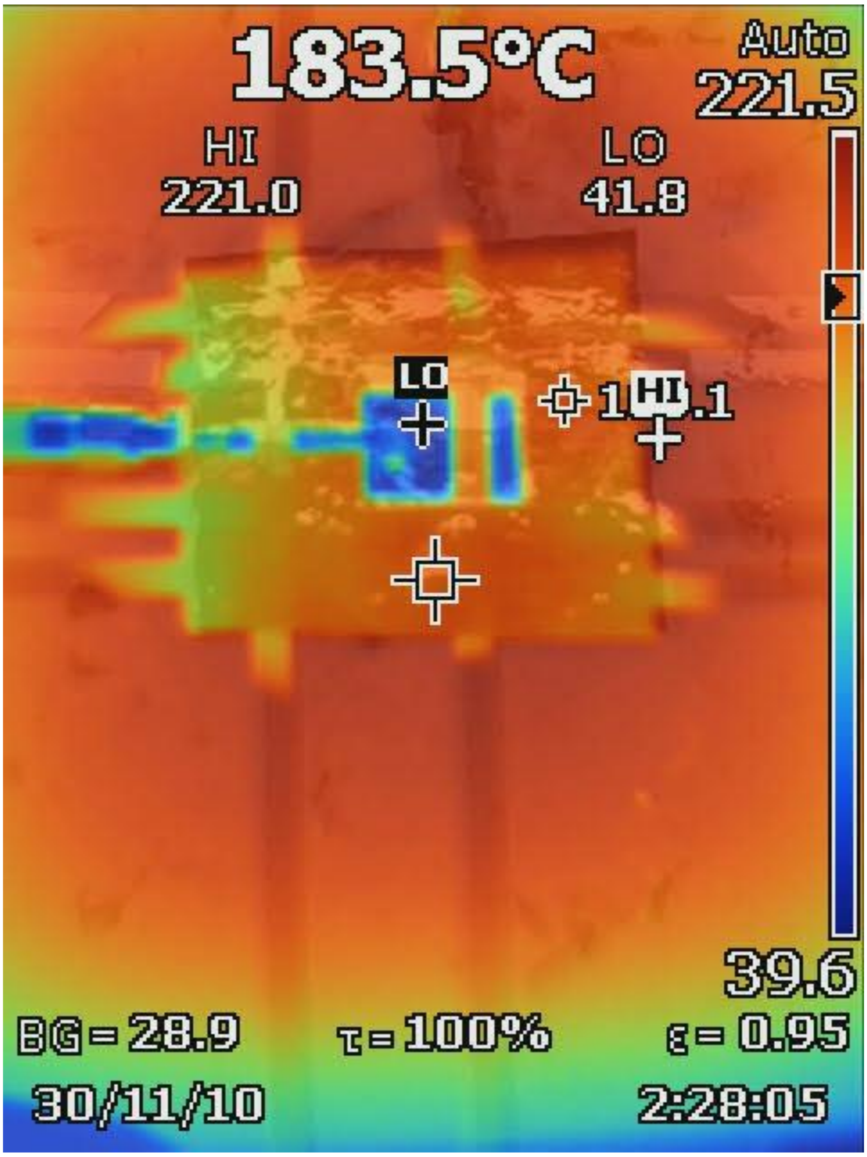

Supplementary Figure 35. Thermal image of the antenna on the hot plate. The 183℃ readout is shown for the temperature at the surface of the composite substrate.

Supplementary Note 19: Anechoic Radiation Pattern Measurements

The radiation patterns were measured using the NSI-2000 scanner shown in Supplementary Figure 36. The hot plate was place on the azimuth turn-table with the elevation scanning arm moved between -/+155°. Given the presence of the hotplate and the antenna's own ground plane, it is anticipated that the radiation through the substrate will be minimal, which was evidenced by the measured patterns in Figure 4(d).

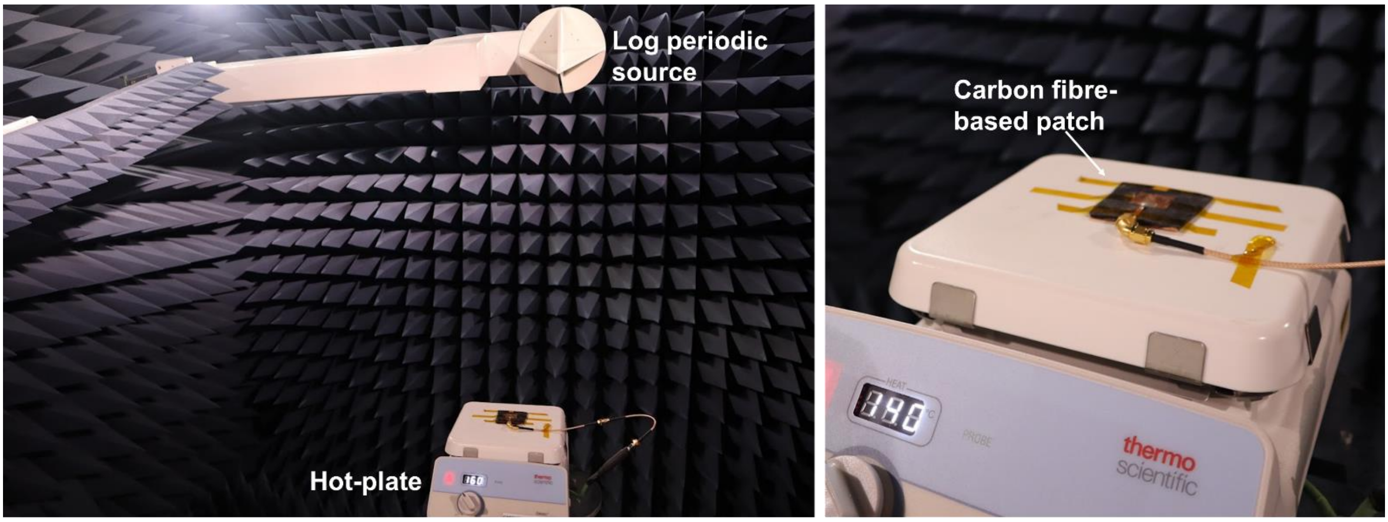

(a) (b)

Supplementary Figure 36. (a) Measurement setup of the thermistor-based patch in the anechoic chamber for obtaining the near-field scans; (b) zoomed-in photo of the antenna on the pre-heated plate.

As the antenna exhibits a wide S11<-6 dB bandwidth due to the high loss in the substrate, the antenna's patterns were measured over different frequencies, for both the room temperature and the high-temperature cases. In both setups the patterns are normalized to the maximum measured value. All patterns were measured in the co-polarized (vertical) direction of the patch's radiation.

Supplementary Figure 37 shows the measured patterns at different frequencies between 2 and 2.6 GHz, in addition to the 2.4 GHz band results shown in Figure 4. At all temperatures, even those where the antenna is mismatched, the temperature sensitivity is very clear with at least 5 dB gain increase. The results shown in the supplementary figure were not normalized and represent the measured channel gain between the log periodic source and the thermistor patch.

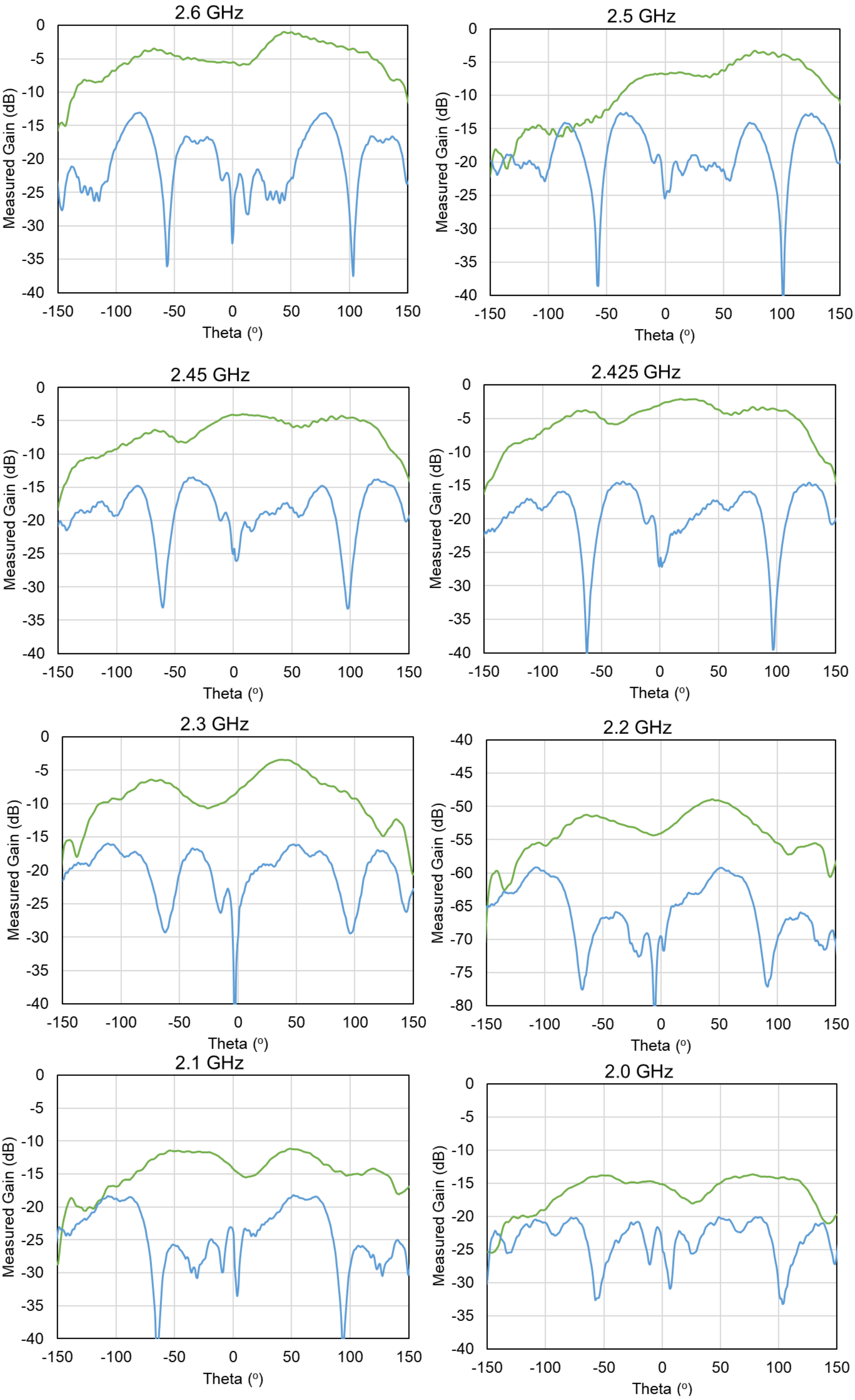

Supplementary Figure 37. measured realized gain patterns between 2 and 2.6 GHz at 20ºC (blue) and 160ºC (green), between the source and the thermistor.

Supplementary Note 20: Far-Field Temperature Sensitivity Characterisation

The channel gain sensory response was evaluated in an echoic indoor environment, shown in Supplementary Figure 38. The compact VNA was used to record the S21 between the horn and the thermistor patch; the broadband response, showing the sensor's operational bandwidth, is shown in Supplementary Figure 39, where the sensor's gain-temperature relation is maintained over 50 MHz of bandwidth.

For the distance variation, only the horizontal distance between the horn and the patch was varied, leading to a varying angular alignment, and the thermistor moving into the horn's side-lobes as the horn approaches the thermistor. The channel gain is shown in Supplementary Figure 40, across the horn's full bandwidth up to 4 GHz, where it can be clearly seen that the rise in temperature can be detected across all distances and almost across the full interrogation bandwidth.

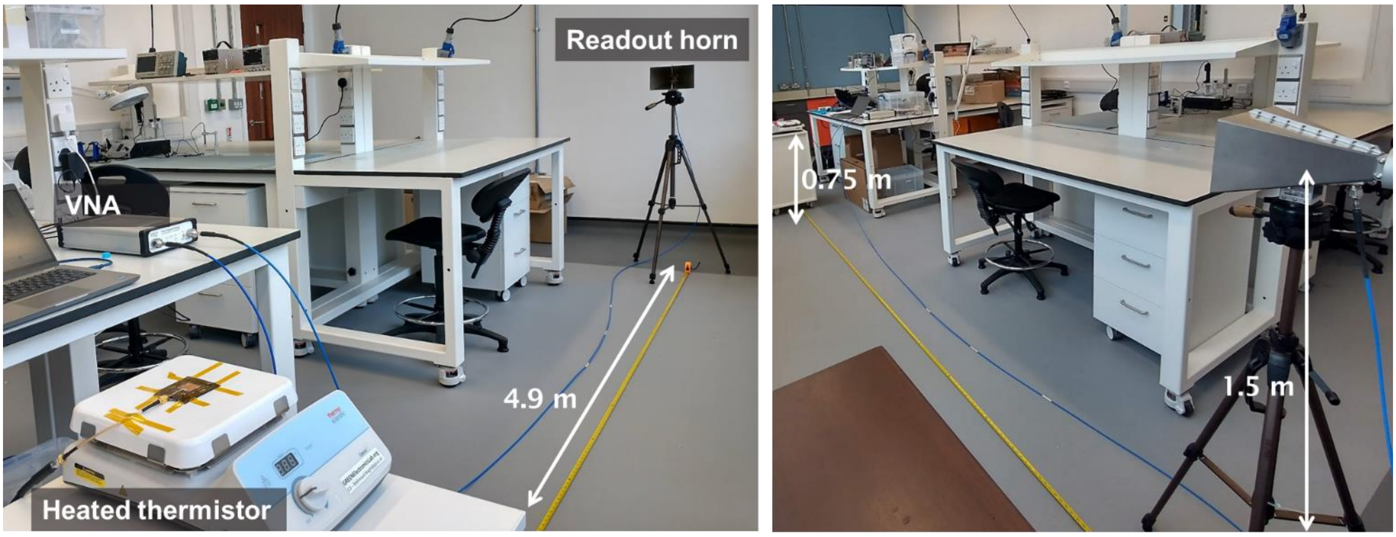

Supplementary Figure 38. Measurement setup of the far-field channel gain at different temperatures.

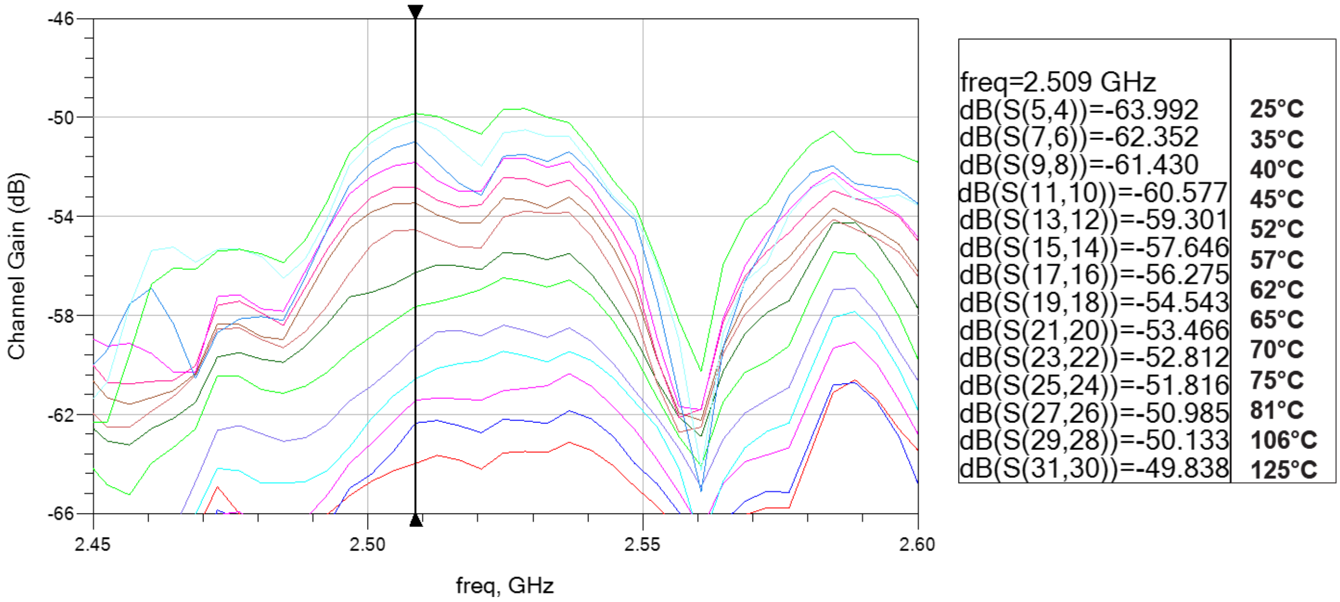

Supplementary Figure 39. Broadband channel gain around the antenna's interrogation frequency, 2.509 GHz, showing a bandwidth of approximately 50 MHz (from 2.5 to 2.55 GHz) over which the gain/temperature relation is maintained.

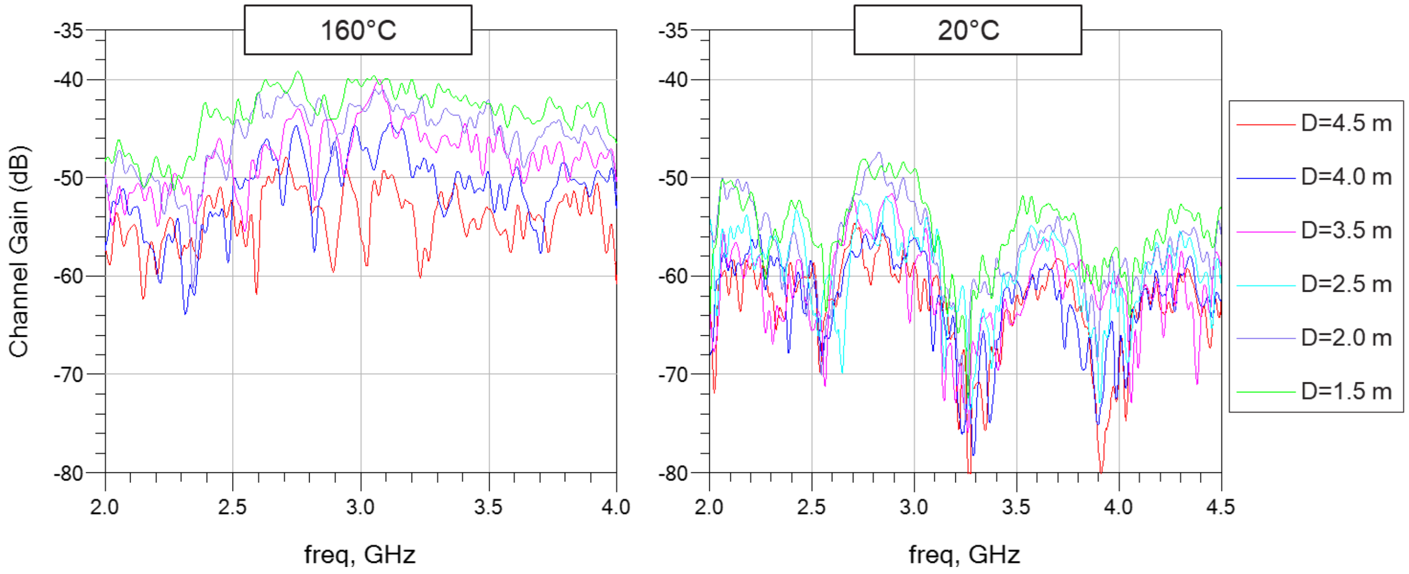

Supplementary Figure 40. Broadband channel gain between the thermistor and horn for varying distances.

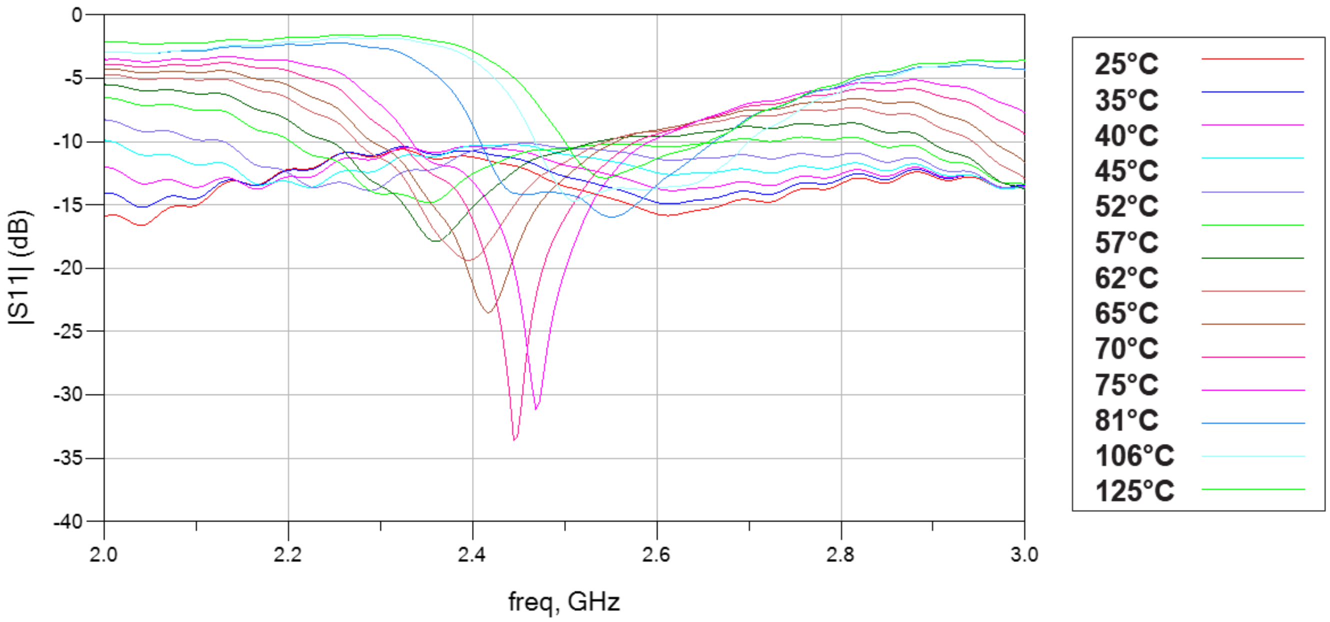

Supplementary Figure 41. Measured reflection coefficient of the patch antenna as a function of temperature, in the same setup used for the channel gain measurements.

It can be observed in Supplementary Figure 41 that the resonance shift moves from 2.0 GHz to approximately 2.6 GHz. This is attributed to the permittivity change previously observed in the microstrip line, in Supplementary Figure 20. The exact resonance frequency of the patch is expected to vary based on its operation environment and also fabrication tolerances in both the composite and the conductor attachment. Nevertheless, the observed sensitivity is comparable to that of the open-ended non-radiative resonators (Resonators 1 and 2), as compared in Figure 4(g).

From the S11 results, obtained in the same setup, the frequency sensitivity of the microstrip patch antenna was evaluated. Supplementary Figure 42 shows the change in the resonant frequency as a function of temperature. For the temperatures over which the antenna's response did not exhibit a clear narrow-band resonance, i.e., T>81ºC in Supplementary Figure 40, the resonance frequency was estimated as the centre frequency of the -10 dB bandwidth of the antenna. As such variations in the S11 are an artefact of the measurement setup, these are included in Supplementary Figure 42 and in the sensitivity calculations, to allow the system to be evaluated in a real environment.

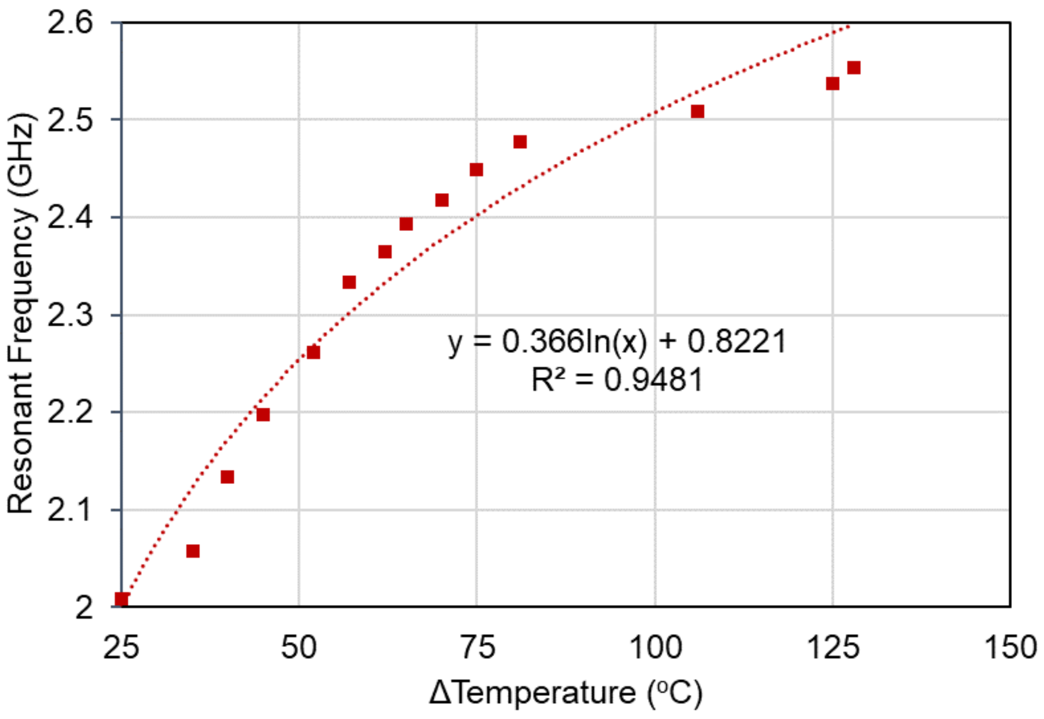

Supplementary Figure 42. resonant frequency of the patch antenna in the hot plate/echoic measurement setup from Supplementary Figure 23.

Supplementary Note 21: RFID RSSI Repeatability Across Channels

The RFID band spans several channels. Therefore, the sensor might be interrogated in any of the channels within the RFID band (915 MHz). The thermistor-coated RFID tag was interrogated at 3 frequencies, 900, 912, and 927 MHz, all falling within the US RFID band. An Impinj RFID reader with a circularly-polarized microstrip patch antenna was used to read the tag's response over the intended band.

Supplementary Figure 43 shows the normalized RSSI changes in the tag's response across the three RFID channels. It can be seen that the same trend is observed with approximately 0.5 dB/^{o}C sensitivity, meaning that a temperature sensing tag could be read across all RFID channels, allowing multiple tags to be read simultaneously in a cluttered environment.

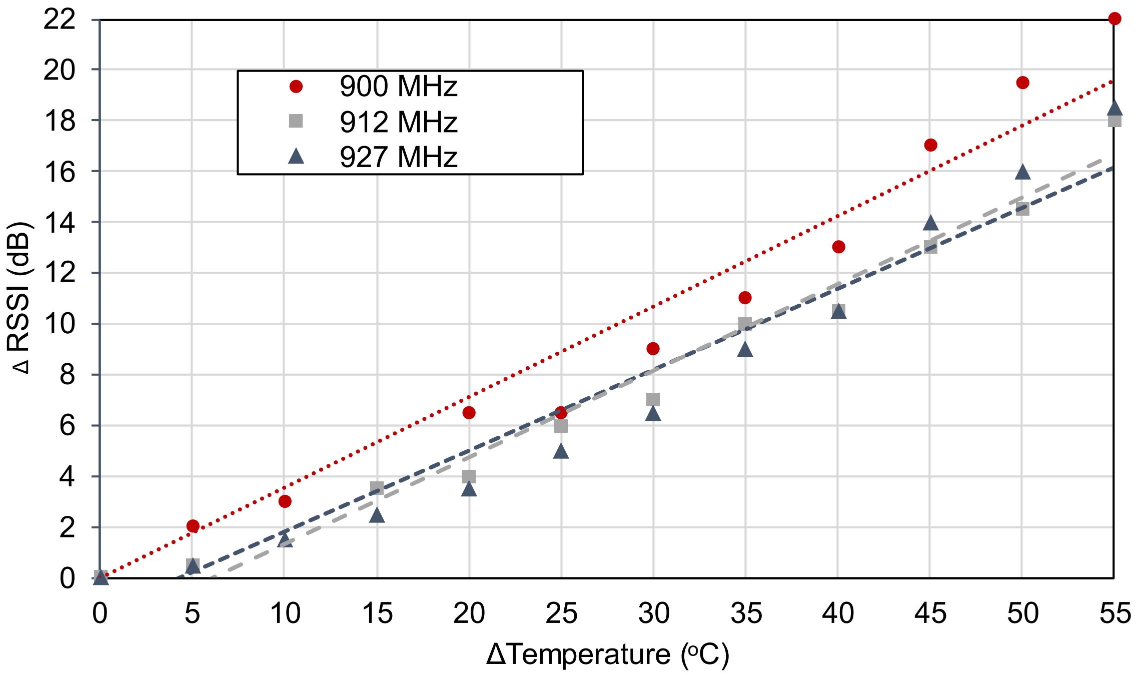

Supplementary Figure 43. normalised RSSI change of the RFID tags in response to temperature changes at different frequencies.

RFID temperature sensors are commercially available using solid-state temperature sensors with on-chip readouts. Examples of such systems include the "WISP" (a research prototype) [S28] and the EM4325 UHF RFID IC (commercial). These systems are fundamentally different from our approach. In both systems, the temperature sensor's response is digitized, converted to digitally-stored data, then digitally modulated in the RFID tag's ID. As a result, such ICs require further functionalities including standard ADCs and/or digital peripherals.

On the other hand, the proposed sensor does not require any analogue-to-digital interface on the tag/sensor side. The resistance changes due to temperature are passively amplitude-modulated onto any wireless signal, and are not limited to specific bands or modulations. Therefore, the proposed sensor could in principle be included in a very simple single-transistor continuous-wave/on-off keying (OOK) modulator, without the need for complex digital circuitry. This makes it valuable in flexible, sustainable, and large-area electronics, where individual RF transistors or diodes have been demonstrated [S29], but large-scale integration (VLSI) remains challenging.

Supplementary References

[S1] Komolafe, A., Torah, R., Wei, Y., Nunes-Matos, H., Li, M., Hardy, D., Dias, T., Tudor, M., & Beeby, S., Integrating flexible filament circuits for e-textile applications. Advanced Materials Technologies, 4(7), 1900176. https://doi.org/10.1002/admt.201900176

[S2] Gupta, N.S.; Lee, K.-S.; Labouriau, A. Tuning Thermal and Mechanical Properties of Polydimethylsiloxane with Carbon Fibers. Polymers 2021, 13, 1141. https://doi.org/10.3390/polym13071141

[S3] Ren, X., et al., A low-operating-power and flexible active-matrix organic-transistor temperature-sensor array. *Advanced materials*, 2016. 28(24): p. 4832-4838.

[S4] Awasthi, C., et al. *Temperature sensing performance of 2D-MoSe2 based thermistor*. in *AIP Conference Proceedings*. 2020. AIP Publishing LLC.

[S5] Wu, J., et al., *Self-Calibrated, Sensitive, and Flexible Temperature Sensor Based on 3D Chemically Modified Graphene Hydrogel.* Advanced Electronic Materials, 2021. 7(4): p. 2001084.

[S6] Kim, Y.-J., et al., *Wood-based flexible graphene thermistor with an ultra-high sensitivity enabled by ultraviolet femtosecond laser pulses.* CIRP Annals, 2021.

[S7] Lee, J.-W., et al., *High sensitivity flexible paper temperature sensor and body-attachable patch for thermometers.* Sensors and Actuators A: Physical, 2020. 313: p. 112205.

[S8] Barmpakos, D., et al., A fully printed flexible multidirectional thermal flow sensor. *Flexible and Printed Electronics*, 2020. 5(3): p. 035005.

[S9] Shin, J., et al., Sensitive wearable temperature sensor with seamless monolithic integration. *Advanced Materials*, 2020. 32(2): p. 1905527.

[S10] Trudeau, C., et al., All inkjet-printed perovskite-based bolometers. *npj Flex Electron*, 2020. 4(1): p. 1-5.

[S11] Su, Y., et al., Printable, highly sensitive flexible temperature sensors for human body temperature monitoring: a review. *Nanoscale Research Letters*, 2020. 15(1): p. 1-34.

[S12] Wang, X., et al., Flexible temperature sensors. *Frontiers in Chemistry*, 2021: p. 780.

[S13] Yokota, T., et al., Ultraflexible, large-area, physiological temperature sensors for multipoint measurements. *Proceedings of the National Academy of Sciences*, 2015. 112(47): p. 14533-14538.

[S14] Hao, S., Fu, Q., Meng, L. et al. A biomimetic laminated strategy enabled strain-interference free and durable flexible thermistor electronics. *Nat Commun* 13, 6472 (2022). https://doi.org/10.1038/s41467-022-34168-x

[S15] Liu, H., Du, C., Liao, L. *et al.* Approaching intrinsic dynamics of MXenes hybrid hydrogel for 3D printed multimodal intelligent devices with ultrahigh superelasticity and temperature sensitivity. *Nat Commun* 13, 3420 (2022). https://doi.org/10.1038/s41467-022-31051-7

[S16] Rajzer, I., Menaszek, E., Bacakova, L. et al. In vitro and in vivo studies on biocompatibility of carbon fibres. *J Mater Sci: Mater Med* 21, 2611–2622 (2010). https://doi.org/10.1007/s10856-010-4108-3

[S17] Miranda I, Souza A, Sousa P, et al. Properties and Applications of PDMS for Biomedical Engineering: A Review. *J Funct Biomater.* 2021;13(1):2. 2021 Dec 21. https://doi.org/10.3390/jfb13010002

[S18] B. Kubina, M. Schüßler, C. Mandel, A. Mehmood and R. Jakoby, "Wireless high-temperature sensing with a chipless tag based on a dielectric resonator antenna," *SENSORS*, 2013 IEEE, Baltimore, MD, USA, 2013, pp. 1-4, doi: 10.1109/ICSENS.2013.6688181.

[S19] Lu F, Tan Q, Ji Y, Guo Q, Guo Y, Xiong J. A Novel Metamaterial Inspired High-Temperature Microwave Sensor in Harsh Environments. *Sensors*. 2018; 18(9):2879. https://doi.org/10.3390/s18092879

[S20] Tan, Q., Ren, Z., Cai, T., Li, C., Zheng, T., Li, S., & Xiong, J. (2015). Wireless passive temperature sensor realized on multilayer htcc tapes for harsh environment. *Journal of Sensors*, 2015, e124058. https://doi.org/10.1155/2015/124058

[S21] Karim, H., Delfin, D., Chavez, L. A., Delfin, L., Martinez, R., Avila, J., Rodriguez, C., Rumpf, R. C., Love, N., & Lin, Y. (2017). Metamaterial based passive wireless temperature sensor. *Advanced Engineering Materials*, 19(5), 1600741. https://doi.org/10.1002/adem.201600741

[S22] Tchafa, F. M., & Huang, H. (2018). Microstrip patch antenna for simultaneous strain and temperature sensing. *Smart Materials and Structures*, 27(6), 065019. https://doi.org/10.1088/1361-665X/aabd47

[S23] Leier, B., Baghelani, M., & Iyer, A. K. (2022). A microwave stripline ring resonator sensor exploiting the thermal coefficient of dielectric constant for high-temperature sensing. *IEEE Sensors Journal*, 22(22), 21666–21675. https://doi.org/10.1109/JSEN.2022.3210779

[S24] Lin, J.-H., & Kao, Y.-H. (2008). Wireless temperature sensing using a passive RFID tag with film bulk acoustic resonator. 2008 *IEEE Ultrasonics Symposium*, 2209–2212. https://doi.org/10.1109/ULTSYM.2008.0547

[S25] Idhaiam, K. S. V., Pozo, P. D., Sabolsky, K., Sabolsky, E. M., Sierros, K. A., & Reynolds, D. S. (2021). All-ceramic lc resonator for chipless temperature sensing within high temperature systems. *IEEE Sensors Journal*, 21(18), 19771–19779. https://doi.org/10.1109/JSEN.2021.3094406

[S26] Cheng, H., Ren, X., Ebadi, S., Chen, Y., An, L., & Gong, X. (2015). Wireless passive temperature sensors using integrated cylindrical resonator/antenna for harsh-environment applications. *IEEE Sensors Journal*, 15(3), 1453–1462. https://doi.org/10.1109/JSEN.2014.2363426

[S27] Zhu, Y., Zheng, Y., Gao, Y., Made, D. I., Sun, C., Je, M., & Gu, A. Y. (2015). An energy autonomous 400 mhz active wireless saw temperature sensor powered by vibration energy harvesting. *IEEE Transactions on Circuits and Systems I: Regular Papers*, 62(4), 976–985. https://doi.org/10.1109/TCSI.2015.2402937

[S28] Liu, G., Yu, R., Liu, D., Xia, Y., Pei, X., Wang, W., Min, C., Liu, S., Shao, R., & Xu, Z. (2022). 3D-printed TiO2-Ti3C2Tx heterojunction/rGO/PDMS composites with gradient pore size for electromagnetic interference shielding and thermal management. *Composites Part A: Applied Science and Manufacturing, 160*, 107058. https://doi.org/10.1016/j.compositesa.2022.107058

[S29] A. P. Sample, D. J. Yeager, P. S. Powledge, A. V. Mamishev and J. R. Smith, "Design of an RFID-Based Battery-Free Programmable Sensing Platform," in IEEE Transactions on Instrumentation and Measurement, vol. 57, no. 11, pp. 2608-2615, Nov. 2008, doi: 10.1109/TIM.2008.925019.

[S30] Georgiadou, D. G., Semple, J., Sagade, A. A., Forstén, H., Rantakari, P., Lin, Y.-H., Alkhalil, F., Seitkhan, A., Loganathan, K., Faber, H., & Anthopoulos, T. D. (2020). 100 GHz zinc oxide Schottky diodes processed from solution on a wafer scale. Nature Electronics, 3(11), 718–725. https://doi.org/10.1038/s41928-020-00484-7
